# Heat transfer enhancement of control nanofluid flow induced by circular porous slider

**DOI:** 10.1186/s11671-026-05061-z

**Published:** 2026-11-03

**Authors:** Qurrath Ul Aine, R Naveen Kumar, M. C. Jayaprakash, Mohit Sahni, R. J. Punith Gowda, Harikaranahalli Puttaiah Shivaraju

**Affiliations:** 1 Computational Science Lab, Department of Mathematics, Amrita School of Engineering, Amrita Vishwa Vidyapeetham, Bengaluru, 560035 India; 2 Department of Information Technology, University of Technology and Applied Sciences, Al Musanna, Sultanate of Oman; 3 Department of Physics, Sharda University, Knowledge Park III, Greater Noida, India; 4 Department of Mathematics, JSS Science and Technology University, Mysuru, 570006 India; 5 Department of Environmental Sciences, School of Life Sciences, JSS Academy of Higher Education and Research, Mysuru, 570015 India; 6 International Center of Environment and Sustainability (ICESu), JSS Academy of Higher Education and Research, Mysuru, 570015 India

**Keywords:** Casson fluid, Circular porous slider, Magnetic field, Thermophoresis and Brownian motion, Active and passive control of nanoparticles

## Abstract

To improve heat and mass transfer efficiency, nanofluid-based thermal engineering needs efficient nanoparticle control mechanisms. Brownian motion and thermophoresis significantly affect heat and mass transfer characteristics and nanoparticle movement. Inspired by these applications, the current work examines the flow and transfer characteristics of a Casson nanofluid moving via a circular porous slider with an applied magnetic field, Brownian motion, thermophoresis and active/passive nanoparticle control. The present research​ compares active and passive nanoparticle control techniques and investigates the coupled interaction of these mechanisms within a non-Newtonian Casson fluid. The governing nonlinear equations are transformed using appropriate similarity transformations into a system of ordinary differential equations, which are then numerically solved using the Runge-Kutta-Fehlberg fourth-fifth order (RKF-45) technique. Additionally, the transfer properties are analyzed using response surface methodology (RSM) and artificial neural networks (ANNs). The ANN forecasts exhibit outstanding agreement with the numerical data, while the RSM model achieves prediction accuracies of 99.48 and 99.88% for the active and passive cases, respectively. Higher temperature and concentration profiles are produced by active nanoparticle control than by passive control. The results are pertinent to enhanced cooling technologies, biomedical transfer systems, porous lubrication devices and thermal energy management. They also offer new perspectives on nanoparticle transfer processes.

Clinical trial: Not applicable.

## Introduction

Casson fluid is a type of non-Newtonian fluid that has a characteristic known as yield stress, which means it only starts flowing when a certain level of stress is exceeded. Before reaching the yield stress level, it acts as a solid but begins to flow like any other fluid after passing the threshold level. As opposed to Newtonian fluids, it shows variation in its viscosity, which is dependent upon the amount of stress imposed on the fluid. Such models have proved very effective in describing the nature of fluids that have some structure inside them, such as blood, chocolate and printing ink. In mathematical modelling, the Casson model uses a parameter that can accommodate the yield stress phenomenon. It finds wide applications in many problems dealing with boundary layer flow and heat transfer, particularly where the realistic physical phenomena of fluid flows must be taken into account. El-ashhab et al. [1] studied the impact of nanoparticle aggregation and entropy generation on stagnation point Casson nanofluid flow through a porous medium. Kashif et al. [2] deliberated entropy generation in two-phase Casson nanofluid flow with microorganisms through a vertical stretching sheet in a porous medium. Ramasekhar et al. [3] studied the impact of rheology on heat transfer, wall shear stress and hemodynamics while studying Casson hybrid nanofluid flow via stenotic arteries. Jagadeesha et al. [4] analyzed stagnation-point flow and heat transfer of Casson hybrid nanofluid. Kumar and Gupta [5] explored the flow of hybrid nanofluids, highlighting an increase in heat transfer using water as the base fluid with nanoparticles of various shapes.

A circular porous slider with fluid suction or injection possible at the interface is used to depict a moving porous surface. This configuration is crucial for controlling boundary layer growth, reducing drag and enhancing heat and mass transfer characteristics. Flow symmetry and velocity distribution are impacted by radial variations brought on by the circular form. By affecting the momentum exchange, porosity is essential in altering the behavior of nearby fluids. They are commonly used in the fields of filtration, biomedical devices and lubrication, where regulated permeability and surface flow are used to maximize stability and performance. With the inclusion of the Casson nanofluid model in the analysis, there are possibilities to account for both the yield stress phenomenon and improved thermal conductivity. Porosity controls the boundary layer thickness, whereas the Casson parameter determines flow resistance and the addition of nanoparticles improves heat transfer capabilities. Wang et al. [6] studied fluid flow past a circular porous slider, where a reduction in the width of the gap results in greater lift and drag, and solutions were achieved using asymptotic, series and numerical approaches. Gorla [7] investigated porous circular slider flow and found that the Reynolds number, injection and Prandtl number have an impact on the velocity and temperature field. Ghoreishi et al. [8] used a one-step optimal homotopy analysis method for the study of circular porous slider flow, where the Reynolds number and injection have a major effect on velocity and viscous dissipation. Faraz et al. [9] examined the influence of magnetism on circular porous slider flow, proving that the transverse magnetic field affects the characteristics of drag and lift of the slider. Singh et al. [10] explored a hybrid double-layered circular porous journal bearing, indicating that pseudoplastic lubricant and roughness contribute to stability and film thickness.

The magnetic field impacts the electrically conductive fluid through the development of the Lorentz force, which acts against the flow and decreases the velocity. The Lorentz force impact on the Casson nanofluid is relatively significant owing to the presence of yield stress and varying viscosity. Additionally, the magnetic field tends to increase the thickness of the momentum boundary layer but reduces the velocity profile. In a circular porous slider, the porous nature enables either suction or injection of the fluid flow. Kattimani et al. [11] investigated the effect of a magnetic field on rotating disk flow, finding that higher levels of magnetization reduce the velocity of the lower disk flow and increase the characteristics of the upper disk flow. Perumalsamy et al. [12] examined the effects of the magnetic field on the flow of Sutterby hybrid nanofluid, which exhibited a reduction in velocity because of the stronger Lorentz force and magnetic damping. Sharma et al. [13] explored the magnetohydrodynamic (MHD) flow of tangent-hyperbolic nanofluid over inclined conical disks, revealing that magnetic field effects greatly affect the flow and heat transfer behavior. Kaur et al. [14] inspected the effect of the magnetic field on the MHD flow of nanofluid over a rotating disk. Kumar and Gupta [15] performed an analysis of MHD flow past a radially stretching sheet, which demonstrated that the magnetic field has an effect on velocity and improves the heat transfer properties of hybrid nanofluids.

The forces that cause a nanoparticle to transition from a hot to a cooler region in a fluid with a temperature gradient are known as thermophoresis. Brownian motion is the random movement of nanoparticles brought on by molecular collisions. For Casson nanofluids, both mechanisms control the transfer of the nanoparticles, with thermophoresis causing them to move in a certain direction and Brownian motion increasing the mixing of the particles and heat transfer. A magnetic field will impose a drag on the fluid flow and will alter the temperature, which has an indirect effect on both mechanisms. The suction and injection effect of velocity, concentration and thermal gradient on a circular porous slider is also modified by the porosity. These effects are important in controlling heat and mass transfer in complex fluid flow systems. Shaaban et al. [16] studied heat transfer enhancement and nanoparticle transfer in Boger nanofluid flow using Brownian motion and thermophoresis effects. Asaigeethan et al. [17] investigated the impact of thermophoresis and Brownian motion on the velocity, temperature and concentration profiles of nanofluid flow over a stretching cylinder. Kumar et al. [18] reviewed the effects of thermophoresis and Brownian motion on bioconvective nanofluids, emphasizing their significance for particle transfer, concentration distributions and heat transfer. Ganji et al. [19] examined the microbe transport properties while studying Brownian motion and thermophoresis effects in MHD flow of penta-hybrid nanofluid. Karthik et al. [20] analyzed the effects of Brownian motion and thermophoresis in Sisko hybrid nanofluid flow over a stretched cylinder for biomedical drug-delivery applications.

**Fig. 1:**
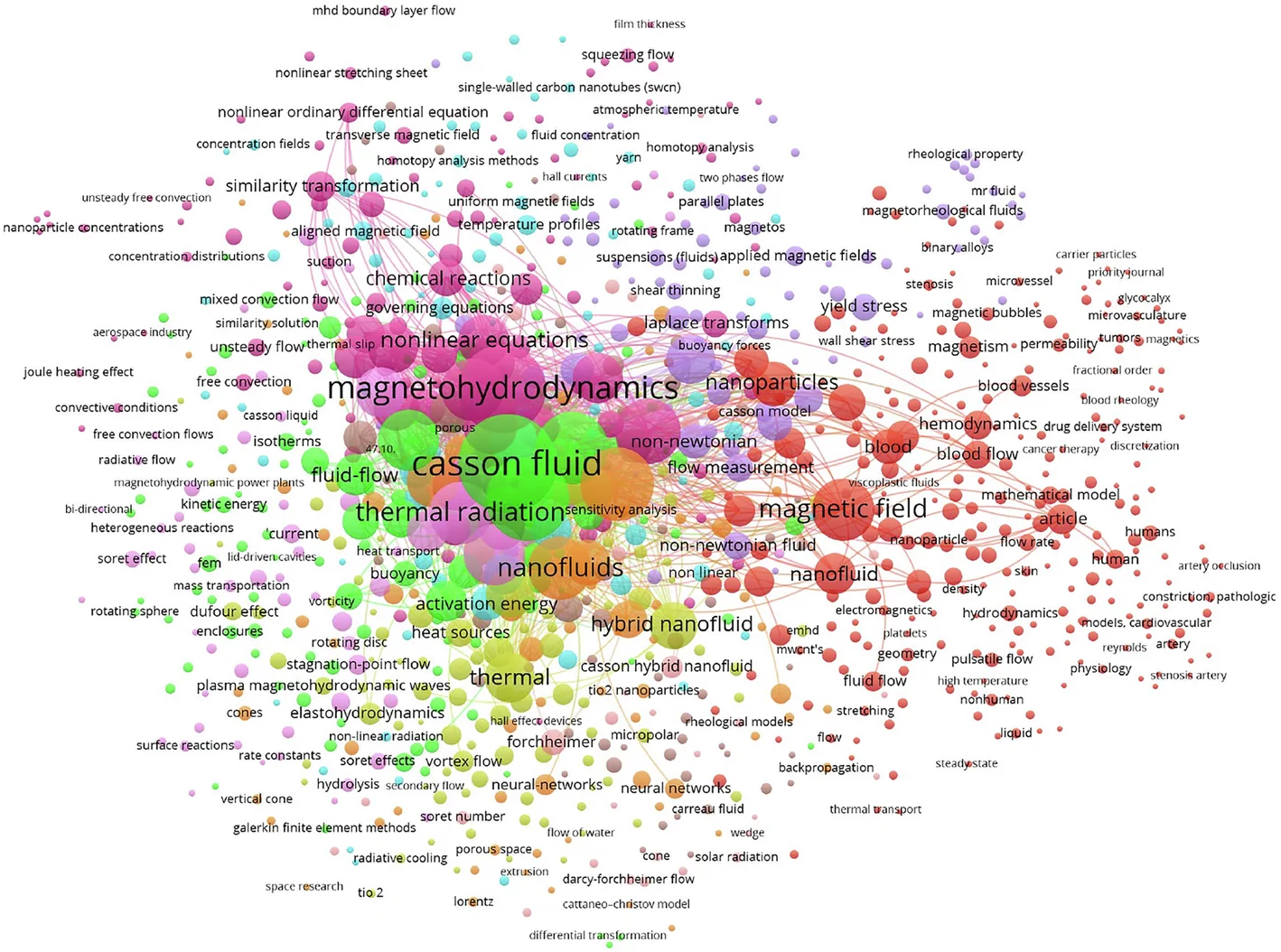
Keyword co-occurrence of the Casson fluid and magnetic field

Active and passive control are two methods of controlling nanoparticle movement in fluid flow. With active control, the concentration of the nanoparticles on the surface is kept constant, whereas with passive control, the particles move naturally based on thermophoresis and Brownian motion. Such mechanisms affect the distribution of particles as well as yield stress effects in a Casson nanofluid. The presence of a magnetic field also reduces fluid velocity and causes a change in temperature and concentration fields, which influence thermophoretic and Brownian motion, respectively. All these effects contribute significantly to the control of heat and mass transfer behavior when interacting. Chebaane et al. [21] investigated physics-informed neural networks to study ternary nanofluid in the analysis of active and passive control of flow involving the cone-disk geometry with consideration of Brownian motion and thermophoresis. Kumar et al. [22] examined active and passive nanoparticle control mechanisms in Boger fluid flow. Bag and Kundu [23] analyzed MHD flow of nanofluid over a thin needle, both with active and passive control and found that thermophoresis and Brownian motion influence the heat and mass transfer. Ahmad et al. [24] studied the effect of nanoparticles on heat transfer over a bidirectional stretching surface with active and passive control of nanoparticles by considering Brownian motion and thermophoresis effects. Kumar and Gupta [25] inspected heat transfer in MHD flow of hybrid nanofluid, showing enhanced heat transfer as the nanoparticle volume fraction increased and as thermal conductivity varied. The keyword co-occurrence analysis shows that the term “Casson nanofluid” is connected with “magnetic field” as shown in Fig. 1, which suggests its importance in the field of fluid dynamics and heat transfer. The network visualization reveals strong connections between these keywords and other related terms like heat transfer, Brownian motion, thermal radiation, entropy generation, porous medium and hybrid nanofluid, indicating the multidisciplinary approach of the research field. From Fig. 1, the larger node size and higher connection strength imply that the flow behavior of Casson nanofluid under the effect of magnetic forces is a topic of interest among many researchers. The clustering pattern also indicates that the applications of magnetic fields to control the velocity, the temperature profile and the thermal conductivity of non-Newtonian nanofluids are widely studied. Furthermore, the analysis indicates an increased focus on engineering and biomedical applications such as cooling technologies, drug delivery systems and the field of industrial thermal management. The overall trend is that the integration of magnetic field effects with models of Casson nanofluid has been confirmed, and it has become a major research direction that is rapidly growing.

The investigation of Casson nanofluid flow across a circular porous slider under the combined effects of an applied magnetic field, Brownian motion, thermophoresis and active/passive nanoparticle control mechanisms is what makes this work novel. The combined influence of these phenomena within a non-Newtonian Casson fluid moving through a circular porous slider has not received much attention, despite the fact that these effects have been studied separately in prior research. The comparison of active and passive nanoparticle control systems, which offers an innovative understanding of nanoparticle transfer, concentration profile and thermal properties, is a unique aspect of this work. Additionally, the study offers a data-driven framework for analyzing complex nanofluid systems by combining traditional numerical simulations with Levenberg-Marquardt artificial neural network (LM-ANN) and RSM to estimate and optimize transfer characteristics. The findings advance the scientific knowledge of the interaction of magnetic forces, nanoparticle migration and non-Newtonian fluid behavior, with potential applications in advanced cooling technologies, biomedical transfer systems, porous lubrication devices and thermal energy management. Table 1 illustrates the comparative review for the current research work.

**Table 1:** A comparative review of earlier research and the unique contributions of the present study

Physical Impact	Faraz et al. [9]	Pramanik [26]	Ramesh [27]	Present study
Circular porous slider	Yes	No	No	Yes
Casson fluid	No	Yes	No	Yes
Magnetic field	Yes	No	No	Yes
Thermophoresis and Brownian motion	No	No	Yes	Yes
Activation energy	No	No	Yes	Yes
Active and passive control of nanoparticles	No	No	Yes	Yes
RSM	No	No	No	Yes
LM-ANN	No	No	No	Yes

## Formulation

**Fig. 2:**
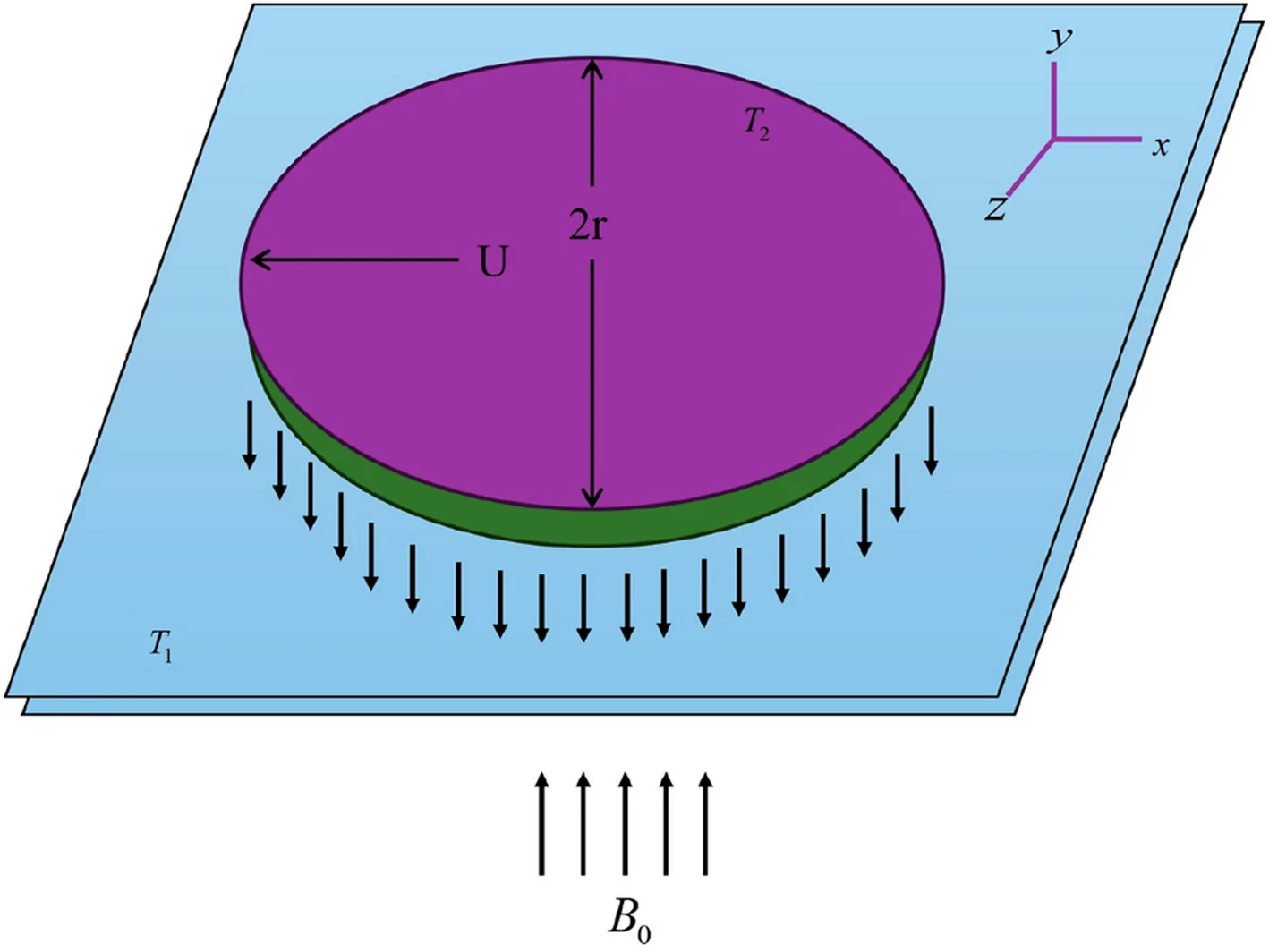
Flow geometry

The flow field caused by a circular porous slider is formulated in this research. The physical arrangement of a three-dimensional nanofluid flow through the circular porous slider is shown in Fig. 2. The slider is separated from the ground by forcing a fluid through its porous bottom. The slider is at a fixed distance, i.e. *z=d*, at the plane, which injects the fluid through it. The nanofluid is forced through the walls of the porous slider at a velocity *W*. The lower plane is located at *z=0* with a velocity *U* in the *x* direction.

The vector form of the Navier-Stokes equations is given as follows:

1 $$\nabla \cdot V=0,$$

2 $$\rho (V\cdot \nabla )V=-\nabla p+\nu {\nabla }^{2}V.$$

Where, *V* is the velocity vector, *ρ * is the density, *ν * is the kinematic viscosity and *p* is the pressure.

3 $$\rho {C}_{p}(V\cdot \nabla )T=k{\nabla }^{2}T,$$

4 $$(V\cdot \nabla )C={D}_{B}{\nabla }^{2}C.$$

Where ${C}_{p}$ is the specific heat, *T* is the temperature, *k* is the thermal conductivity, *C* is the concentration and ${D}_{B}$ is the mass diffusivity.

### Assumptions and conditions of the model

The following are the conditions and assumptions of the current model

- Steady, laminar and incompressible Casson fluid flow.
- Circular porous slider.
- Applied magnetic field, Nanofluid flow.
- Activation energy, thermophoresis and Brownian motion.
- Boundary-layer approximation.
- Active and passive control of nanoparticles.

### Model equation

Under these assumptions, the governing equations describing the flow, heat and mass transfer are formulated as nonlinear partial differential equations (PDEs), which are given as follows [7, 9, 26–29]:

5 $${u}_{x}+{v}_{y}+{w}_{z}=0,$$

6 $$u{u}_{x}+v{u}_{y}+w{u}_{z}=-\frac{1}{{\rho }_{nf}}{p}_{x}+{\nu }_{nf}(1+\frac{1}{\beta })({u}_{xx}+{u}_{yy}+{u}_{zz})-\frac{{\sigma }_{nf}{B}_{0}^{2}}{{\rho }_{nf}}u,$$

7 $$u{v}_{x}+v{v}_{y}+w{v}_{z}=-\frac{1}{{\rho }_{nf}}{p}_{y}+{\nu }_{nf}(1+\frac{1}{\beta })({v}_{xx}+{v}_{yy}+{v}_{zz})-\frac{{\sigma }_{nf}{B}_{0}^{2}}{{\rho }_{nf}}v,$$

8 $$u{w}_{x}+v{w}_{y}+w{w}_{z}=-\frac{1}{{\rho }_{nf}}{p}_{z}+{\nu }_{nf}(1+\frac{1}{\beta })({w}_{xx}+{w}_{yy}+{w}_{zz})--\frac{{\sigma }_{nf}{B}_{0}^{2}}{{\rho }_{nf}}w,$$

9 $$\begin{array}{c}u{T}_{x}+v{T}_{y}+w{T}_{z}=\frac{{\kappa }_{nf}}{{(\rho {C}_{p})}_{nf}}({T}_{xx}+{T}_{yy}+{T}_{zz})\\ \,+\,\tau [{D}_{B}({C}_{x}{T}_{x}+{C}_{y}{T}_{y}+{C}_{z}{T}_{z})+\frac{{D}_{T}}{{T}_{2}}({T}_{x}^{2}+{T}_{y}^{2}+{T}_{z}^{2})],\end{array}$$

10 $$\begin{array}{c}u{C}_{x}+v{C}_{y}+w{C}_{z}={D}_{B}({C}_{xx}+{C}_{yy}+{C}_{zz})\\ \,+\,\frac{{D}_{T}}{{T}_{2}}({T}_{xx}+{T}_{yy}+{T}_{zz})-{k}_{r}^{2}(C-{C}_{2}){(\frac{T}{{T}_{2}})}^{n}\exp (\frac{-{E}_{a}}{\lambda T}).\end{array}$$

The corresponding boundary conditions are as follows [7, 27]:

11 $$\left.\begin{array}{l}u=U,v=0,w=0,T={T}_{1},i)C={C}_{1}({\rm{active}}\,{\rm{control}}),\\ ii)\,{D}_{B}{C}_{z}+\frac{{D}_{T}}{{T}_{2}}{T}_{z}=0({\rm{passive}}\,{\rm{control}}) at\,z=0,\\ u=0,v=0,w=-W,T={T}_{2},C={C}_{2} at\,z=d.\end{array}\right\}$$

At *z=0* the velocity component is $u=U,\,v=0,\,w=0$, which means that the fluid flows in the same way as the circular porous slider in the lateral direction with velocity *U* and the normal velocity is zero. The temperature is kept at $T={T}_{1}$. In active nanoparticle flow control, the concentration of nanoparticles is set to be $C={C}_{1}$ and in passive nanoparticle flow control, ${D}_{B}{C}_{z}+\frac{{D}_{T}}{{T}_{2}}{T}_{z}=0$ signifies that there is zero nanoparticle flux due to Brownian motion and thermophoresis. For *z=d*, the velocity component is *u=v=0*, which is the stationarity condition, while *w=-W* is the fluid speed injected from the porous bottom of the slider. The system is influenced by an externally applied transverse magnetic field. The nanoparticle transfer is modelled using thermophoresis and Brownian motion, incorporating both active control and passive control mechanisms. The combination of the *Cu* and blood as the base fluid is considered. *λ * is the Boltzmann constant, ${k}_{r}^{2}$ is the reaction rate, *(u,v,w)* is the velocity coordinate in the direction of *(x,y,z)*, ${E}_{a}$ is the activation energy, ${B}_{0}$ is the magnetic field strength, *σ * is the electrical conductivity, *β * is the Casson parameter and *W* is the injection velocity.

## Thermophysical properties

Thermophysical properties are physical properties that affect the behaviour of a substance or fluid in response to thermal energy, thermal flow and external forces. In fluid flow and heat transfer problems, these properties are paramount and play an important role in mass transfer, momentum and energy transfer, especially when applications involve nanofluids. The key thermophysical characteristics are as follows [30]:

12 $${\rm{Density}}:{\rho }_{nf}={\varTheta }_{1}{\rho }_{1}+(1-{\varTheta }_{1}){\rho }_{f}.$$

13 $${\rm{Specific}}\,{\rm{heat}}:{(\rho {C}_{p})}_{nf}=(1-{\varTheta }_{1}){(\rho {C}_{p})}_{f}+{\varTheta }_{1}{(\rho {C}_{p})}_{1}.$$

14 $${\rm{Dynamic}}\,{\rm{viscosity}}:{\mu }_{nf}=\frac{{\mu }_{f}}{{(1-{\varTheta }_{1})}^{2.5}}.$$

15 $${\rm{Thermal}}\,{\rm{conductivity}}:\frac{{k}_{nf}}{{k}_{f}}=\frac{{k}_{1}-2{\varTheta }_{1}({k}_{f}-{k}_{1})+2{k}_{f}}{{k}_{1}+{\varTheta }_{1}({k}_{f}-{k}_{1})+2{k}_{f}}.$$

16 $${\rm{Electrical}}\,{\rm{conductivity}}:\frac{{\sigma }_{nf}}{{\sigma }_{f}}=\frac{{\sigma }_{1}-2{\varTheta }_{1}({\sigma }_{f}-{\sigma }_{1})+2{\sigma }_{f}}{{\sigma }_{1}+{\varTheta }_{1}({\sigma }_{f}-{\sigma }_{1})+2{\sigma }_{f}}.$$

### Thermophysical features of the base fluid and nanoparticle

The characteristics of the base fluid and the nanoparticle *Cu* is used in this current research are shown in Table 2.

**Table 2:** The thermophysical characteristics [19,31]

Fundamental properties	Blood (Base fluid)	*Cu*
$$k\times {10}^{2}$$$$(kgm{s}^{-3}{K}^{-1})$$	0.00492	4.0
$${C}_{p}\times {10}^{2}$$$$(Jk{g}^{-1}{K}^{-1})$$	35.94	3.85
$$\rho \times {10}^{2}$$$$(kg{m}^{-3})$$	10.63	89.33
*σ *$({\varOmega }^{-1}{m}^{-1})$	0.8	$5.96\times {10}^{7}$

### Solutions for the PDEs

The similarity transformations are taken in the form as follows:

17 $$\left.\begin{array}{l}u=\frac{W}{d}\mathop{xh}\nolimits^{\prime} (\eta )+Uf(\eta ),v=\frac{W}{d}\mathop{yh}\nolimits^{\prime} (\eta ),\,w=-2Wh(\eta ),\\ \eta =\frac{z}{d},\,\theta (\eta )=\frac{T-{T}_{2}}{{T}_{1}-{T}_{2}},\chi (\eta )=\frac{C-{C}_{2}}{{C}_{1}-{C}_{2}}({\rm{active}}\,{\rm{control}}),\\ \chi (\eta )=\frac{C-{C}_{2}}{{C}_{2}}({\rm{passive}}\,{\rm{control}}),\\ p=-\frac{{\rho }_{f}{W}^{2}K}{2{d}^{2}(t)}({x}^{2}+{y}^{2})-\frac{{\rho }_{f}}{2}{w}^{2}+{\mu }_{f}{w}_{z}-{\rho }_{f}A.\end{array}\right\}$$

Where, *A* and *K* are the constants.

Substituting Eq. (17) into the Eqs. (5) to (10):

18 $$\left(1+\frac{1}{\beta }\right)\frac{{A}_{2}}{{A}_{1}}{h}^{iv}+2\mathop{Rehh}\nolimits^{\prime\prime\prime} -\frac{{A}_{5}}{{A}_{1}}\mathop{Mh}\nolimits^{\prime\prime} =0,$$

19 $$\left(1+\frac{1}{\beta }\right)\frac{{A}_{2}}{{A}_{1}}\mathop{f}\nolimits^{\prime\prime} +2\mathop{Rehf}\nolimits^{\prime} -\mathop{Refh}\nolimits^{\prime} -\frac{{A}_{5}}{{A}_{1}}Mf=0,$$

20 $$\frac{{A}_{3}}{{A}_{4}}\frac{1}{\Pr }{\theta}^{\prime\prime} +2Reh\theta^{\prime} +{N}_{t}{(\theta^{\prime} )}^{2}+{N}_{b}\theta^{\prime} \chi^{\prime} =0,$$

21 $$\chi ^{\prime\prime} +2ReSch{\chi} ^{\prime} -{K}_{r}Sc\chi {(1+{\theta }_{w}\theta )}^{n}\exp \left(\frac{-E}{1+{\theta }_{w}\theta }\right)+\frac{{N}_{t}}{{N}_{b}}\theta ^{\prime\prime} =0.$$

The reduced Boundary conditions are as follows:

22 $$\left.\begin{array}{l}\mathop{h}\nolimits^{\prime} (0)=0,f(0)=1,h(0)=0,\theta (0)=1,\chi (0)=1({\rm{active}}\,{\rm{control}}),\\ {N}_{b}\chi^ {\prime} +{N}_{t}\theta ^{\prime} =0({\rm{passive}}\,{\rm{control}})\,\qquad \,\, at\,\,\eta =0,\\ \mathop{h}\nolimits^{\prime} (1)=0,f(1)=0,h(1)=\frac{1}{2},\theta (1)=0,\,\chi (1)=0\,at\,\eta =1.\end{array}\right\}$$

Description of the non-dimensional parameters:

$\Pr =\frac{{\nu }_{f}{(\rho Cp)}_{f}}{{\kappa }_{f}}$ is the Prandtl number, $Re=\frac{Wd}{{\nu }_{f}}$ is the cross-flow Reynolds number, ${\theta }_{w}=\frac{{T}_{1}-{T}_{2}}{{T}_{2}}$ is the Temperature ratio, ${N}_{b}=\frac{\tau {D}_{B}({C}_{1}-{C}_{2})}{{\nu }_{f}}$ is the Brownian motion parameter for active control of a nanoparticle, ${N}_{b}=\frac{\tau {D}_{B}{C}_{2}}{{\nu }_{f}}$ is the Brownian motion parameter for passive control of a nanoparticle, ${N}_{t}=\frac{\tau {D}_{T}({T}_{1}-{T}_{2})}{{T}_{2}{\nu }_{f}}$ is the Thermophoresis parameter, $Sc=\frac{{\nu }_{f}}{{D}_{B}}$ is the Schmidt number, ${K}_{r}=\frac{{k}_{r}^{2}{d}^{2}}{{\nu }_{f}}$ is the Chemical reaction parameter, $E=\frac{{E}_{a}}{\lambda {T}_{2}}$ is the activation energy parameter and $M=\frac{{\sigma }_{f}{B}_{0}^{2}{d}^{2}}{{\rho }_{f}{\nu }_{f}}$ is the Magnetic field parameter.

### Real-world application of the copper-based nanofluid

**Fig. 3:**
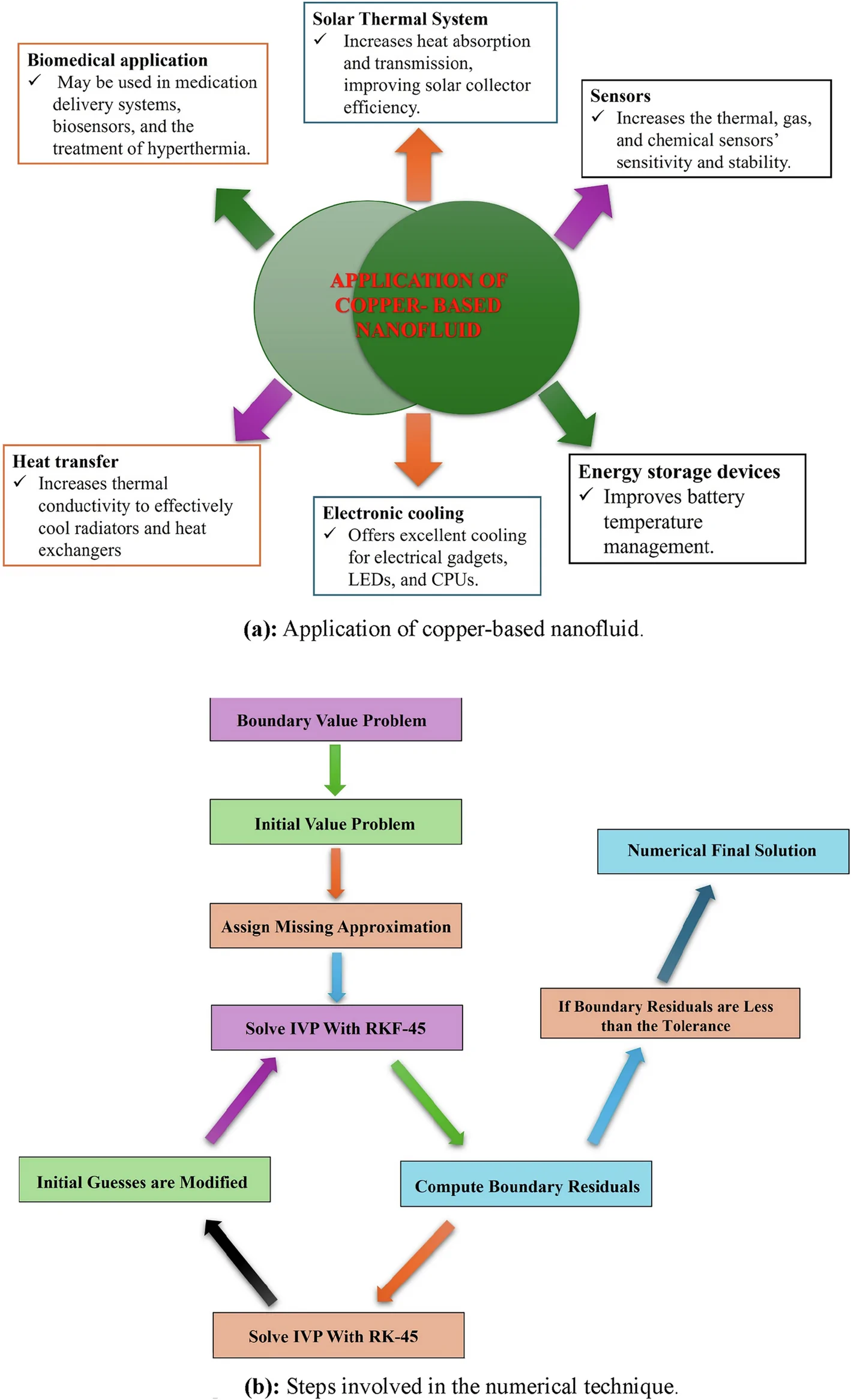
**a:** Application of copper-based nanofluid. **b:** Steps involved in the numerical technique

Nanofluids containing copper nanoparticles have excellent thermal conductivity and stability and as a result, they have numerous applications in engineering and biomedical fields. As regards engineering applications, nanofluids containing copper particles greatly contribute to an increase in the rate of thermal energy transfer in heat exchangers, radiators, cooling pipes and HVAC systems due to high heat dissipation efficiency. As far as biomedical applications are concerned, they are characterised by antimicrobial and therapeutic properties that may promote their use in drug delivery, biosensors, hyperthermia therapy and medical imaging. In solar thermal systems, it may help to boost solar energy absorption and thermal conversion efficiency. It can be used to improve the performance of solar collectors and solar heaters. In sensor applications, nanofluids containing copper particles may help to increase sensitivity, electrical conductivity and detection accuracy in thermal, chemical and gas sensors. In the context of energy storage devices such as batteries and supercapacitors, copper nanofluids help to improve thermal management, energy storage capacity, stability and longevity. In addition, copper nanofluids are great for electronic cooling because they effectively cool electronic devices and power electronics. Figure 3(a) illustrates the application in the real world for the copper-based nanofluid.

## Numerical approach

In the present study, the shooting technique and the RKF-45 approach are both employed. This is a popular and effective numerical technique for solving ordinary differential equations (ODEs), particularly when the differential equations are obtained from a reduction of a more complex PDE. In particular, its adaptive step-size method reduces local truncation error, maintains accuracy and reduces the cost of processing. This method is commonly used in fluid mechanics to study the temperature, concentration and velocity profile of the flow over a flat plate, in a channel and in porous media. When analytical solutions are not possible, its capacity to handle complicated boundary conditions and nonlinearities are very helpful. Figure 3(b) illustrates the steps of the numerical technique. The governing equations and boundary conditions are solved within the computational domain $0\le \eta \le 1$. The RKF-45 shooting method belongs to the category of ODE solvers that use adaptive step sizes. Convergence of the solution has been verified by using step size refinement. The calculations have been carried out using a step size of 0.01 and a convergence criterion of 10^−6^. Convergence of the solution has been achieved when the residual of the prescribed boundary conditions became less than 10^−6^. The step size of 0.01 provides the accuracy and stability of the numerical solution and has been used in all subsequent calculations.

The following presumptions are established in order to initiate the RKF-45 method:

23 $$\left.\begin{array}{c}{y}_{1}=h(\eta ),{y}_{2}=\mathop{h}\nolimits^{\prime} (\eta ),{y}_{3}=\mathop{h}\nolimits^{\prime\prime} (\eta ),{y}_{4}=\mathop{h}\nolimits^{\prime\prime\prime} (\eta ),\\ {y}_{5}=f(\eta ),{y}_{6}=\mathop{f}\nolimits^{\prime} (\eta ),{y}_{7}=\phi (\eta ),{y}_{8}=\phi^ {\prime} (\eta ),\\ {y}_{9}=\chi (\eta ),{y}_{10}=\chi ^{\prime} (\eta ).\end{array}\right\}$$

Substituting Eq. (19) into Eqs. (14) to (18), we obtain

24 $${y}_{4}^{{\prime} }=\left(\frac{{A}_{1}\beta }{({A}_{2}(\beta +1))}\right)\left(\frac{{A}_{5}}{{A}_{1}}M{y}_{3}-2Re{y}_{1}{y}_{4}\right),$$

25 $${y}_{6}^{{\prime} }=\left(\frac{({A}_{1}\beta )}{({A}_{2}(\beta +1))}\right)\left(Re{y}_{5}{y}_{2}-2Re{y}_{1}{y}_{6}+\left(\frac{{A}_{5}}{{A}_{1}}\right)M{y}_{5}\right),$$

26 $${y}_{8}^{{\prime} }=\left(\Pr \frac{{A}_{4}}{{A}_{3}}\right)\left(-2Re{y}_{1}{y}_{8}-{N}_{t}{{y}_{8}}^{2}-{N}_{b}{y}_{10}{y}_{8}\right),$$

27 $$\begin{array}{c}{y}_{10}^{{\prime} }=KrSc{y}_{9}{(1+{\theta }_{w}\theta )}^{n}\exp \left(\frac{-E}{1+{\theta }_{w}\theta }\right)-\frac{{N}_{t}}{{N}_{b}}\\ \,\left(\left(\Pr \frac{{A}_{4}}{{A}_{3}}\right)(-2Re{y}_{1}{y}_{8}-{N}_{t}{{y}_{8}}^{2}-{N}_{b}{y}_{10}{y}_{8})\right)-2\mathrm{Re}Sc{y}_{1}{y}_{10}.\end{array}$$

28 $${y}_{2}(0)=0,{y}_{5}(0)-1=0,{y}_{1}(0)=0,{y}_{7}(0)-1=0,{y}_{9}(0)=0({\rm{active}}\,{\rm{case}}),$$

29 $${y}_{2}(0)=0,{y}_{5}(0)-1=0,{y}_{1}(0)=0,{y}_{7}(0)-1=0,Nb{y}_{10}+Nt{y}_{8}=0({\rm{passive}}\,{\rm{case}}),$$

30 $${y}_{2}(1)=0,{y}_{5}(1)=0,{y}_{1}(1)-0.5=0,{y}_{7}(1)=0,{y}_{9}(1)=0.$$

## Results and discussion

In the present study, different effects such as the magnetic field, thermophoresis, Brownian motion, active and passive control of nanoparticles are considered to explore the heat and mass transfer of the Casson nanofluid. This section provides an insightful explanation for the reasons behind the increasing or decreasing trend for fluid profiles with respect to various parameters. Table 3 shows the comparison of the current work with the published literature for some reduced cases. Table 4 illustrates the baseline parameters used in the recent study.

**Table 3:** Comparison of current work results with published work for some reduced cases

$\mathrm{Re}$	$\mathop{f}\nolimits^{\prime} (1)$		$\mathop{h}\nolimits^{\prime\prime} (0)$		$\mathop{h}\nolimits^{\prime\prime\prime} (0)$	
	Wang [6]	Current work	Wang [6]	Current work	Wang [6]	Current work
0	−1.00000	−1.000000	3.00000	3.000000	−6.0000	−6.000000
0.50043	−0.79655	−0.784513	3.0938	3.081375	−6.3939	−6.383459
2.5895	−0.29382	−0.271547	3.5010	3.498624	−8.1987	−8.187594
5.5727	−0.062841	−0.052493	4.0969	4.086579	−11.129	−11.112381
13.302	−0.0007	−0.000712	5.5060	5.498542	−19.540	−19.524986

**Table 4:** Baseline parameters

Dimensionless parameter	Baseline values
$\mathrm{Re}$	1.5
*β *	0.3
*M*	5
*Sc*	1.0
${\theta }_{w}$	1
${N}_{t}$	0.05
${N}_{b}$	0.3
*E*	1

### Velocity profile

**Fig. 4:**
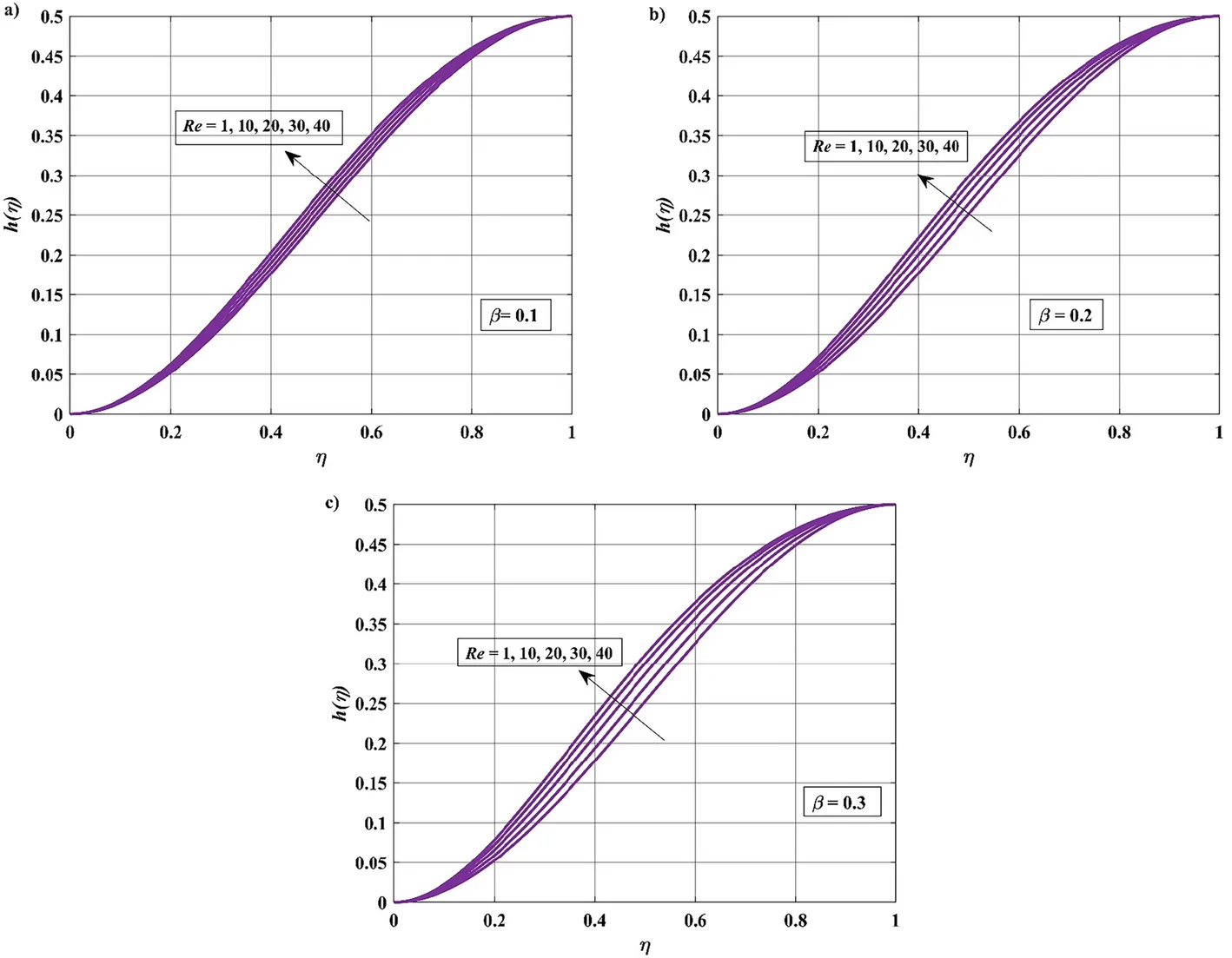
Vertical velocity profile *h(η )* for **a**) *β =0.1*, **b**) *β =0.2*, **c**) *β =0.3*

The influence of the Reynolds number *(Re)* on the *h(η )* is shown in Fig. 4 for various *β *. Figure 4(a) represents the case for *β *= 0.1. The effect of *Re* results in an increase in the *h(η )*, where the close distance between the curves suggests that the effect is not significant for low *β *. In Fig. 4(b) for *β * = 0.2, the effect of *Re* becomes more significant since the increase of the *Re* leads to an increase in the *h(η )*. Figure 4(c) demonstrates the same tendency but for *β *= 0.3. However, the effect of *Re* appears to be much more significant. This is because of the fact that the fluid possesses non-Newtonian properties and therefore an increase in the value of *β * will cause the fluid flow between the surfaces to behave as a non-Newtonian fluid. The viscosity of the fluid increases and the velocity of the fluid for different *Re* becomes significant.

**Fig. 5:**
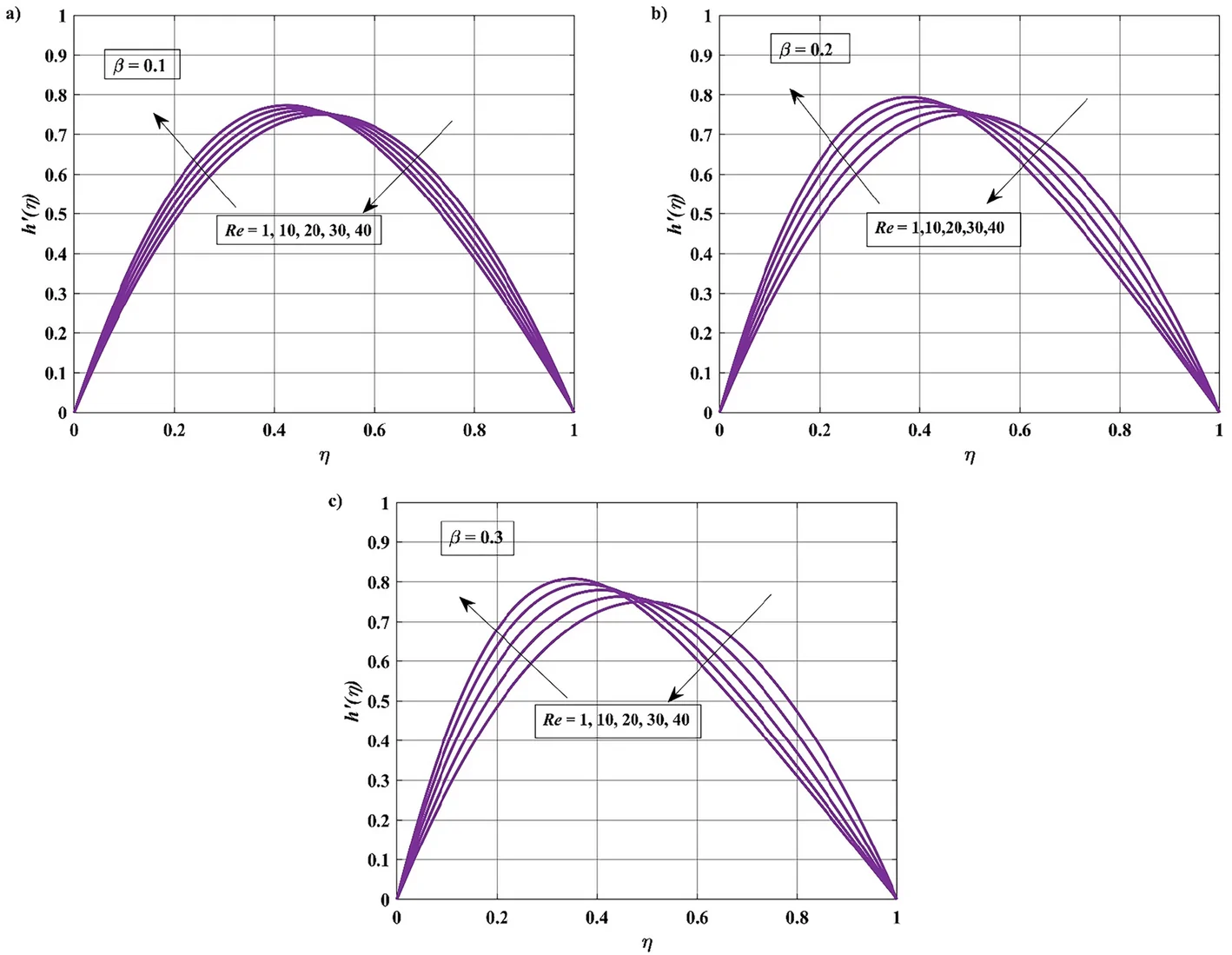
Lateral velocity profile $\mathop{h}\nolimits^{\prime} (\eta )$ for **a**) *β =0.1*, **b**) *β =0.2*, **c**) *β =0.3*

**Fig. 6:**
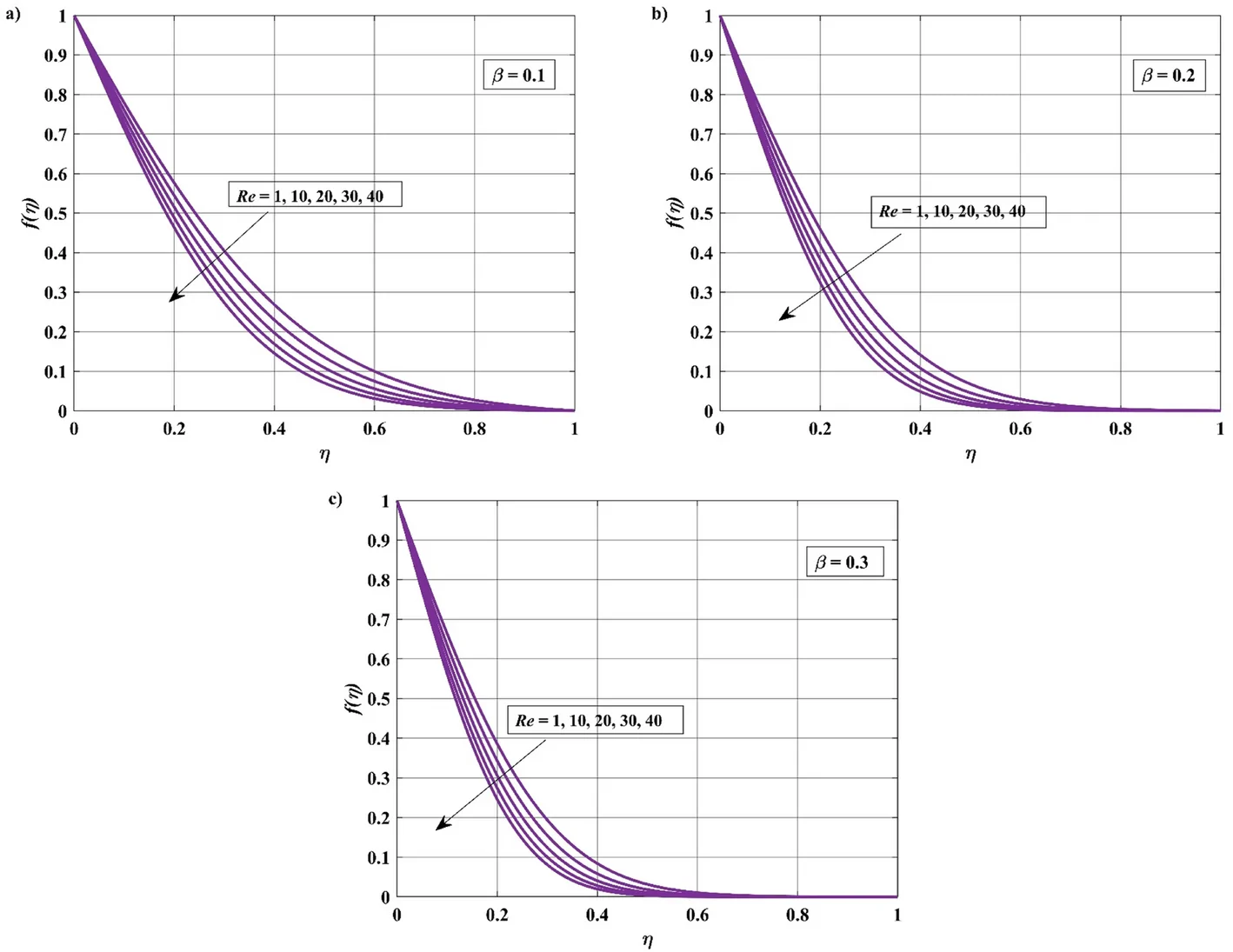
Lateral velocity profile *f(η )* for **a**) *β =0.1*, **b**) *β =0.2*, **c**) *β =0.3*

The lateral velocity profile $\mathop{h}\nolimits^{\prime} (\eta )$ is shown in Fig. 5 for varying *β *. Figure 5(a) illustrates that the $\mathop{h}\nolimits^{\prime} (\eta )$ is parabolic for minimal values of *Re* and becomes more asymmetrical as *Re* rises. As the cross-flow *Re* grows, the amplitude $\mathop{h}\nolimits^{\prime} (\eta )$ rises, but the location of maximum velocity shifts nearer to the moving wall. Figure 5(b) and (c) exhibit a similar trend for varying values of *β *. Figure 6 illustrates that the vertical velocity $\mathop{h}\nolimits^{\prime} (\eta )$ rises in the interval $0\le \eta \le 0.5$ as *Re* increases, whereas it declines in the interval $0.5\le \eta \le 1$. This increasing phenomenon is caused by the momentum and inertia that have been acquired in the flow field because of the increase of *Re*, leading to a higher acceleration of the fluid flow in the upper boundary region. On the other hand, this phenomenon is being suppressed in the lower boundary region. In addition, the greater *β * makes the impact of *Re* more evident.

The lateral velocity profile at the various *Re* of cross-flow, for the chosen Casson parameter (*β *= 0.1; 0.2; 0.3), is shown in Fig. 6(a)–(c). It is noted that the velocity profile monotonically decreases from the moving wall to the stationary wall, with the prescribed boundary conditions being satisfied. As *Re* increases, the magnitude of *f(η )* decreases considerably in the domain of the flow. This is because of the greater cross-flow and inertial effects with the increasing cross-flow *Re* that act against the main flow and lower the lateral velocity. A comparison between Fig. 6(a)–(c) indicates that as *β * increases, the effect of *Re* increases and improves the momentum interaction between the fluid system.

**Fig. 7:**
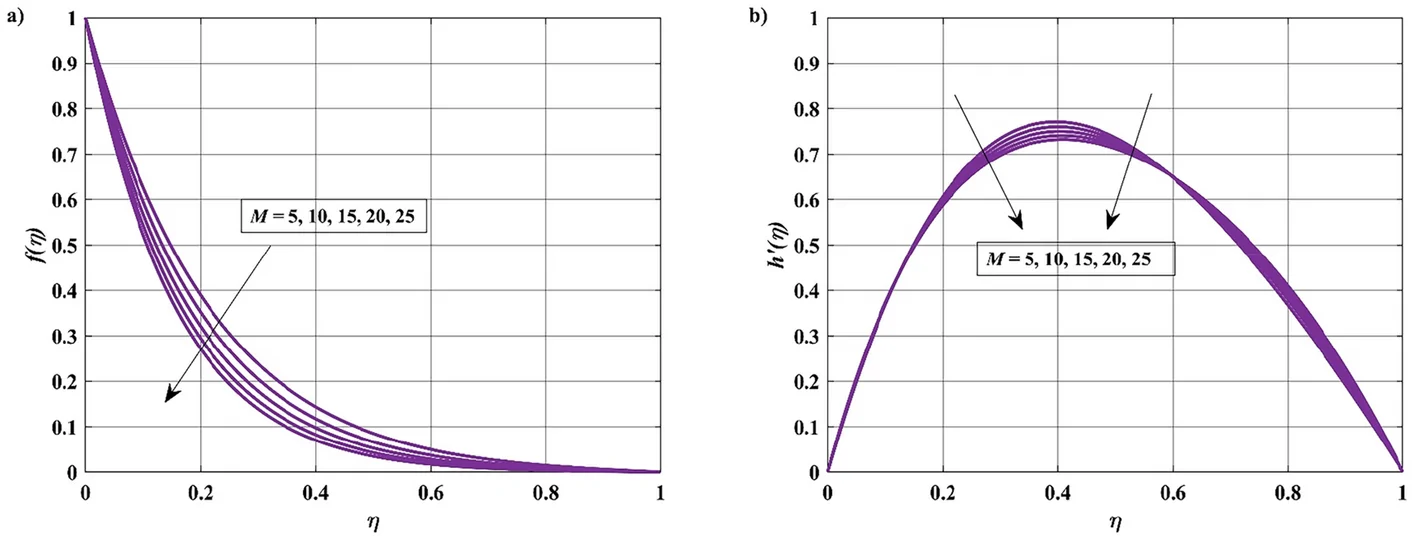
Impact of *M* on **a**) *f(η )*, **b**) $\mathop{h}\nolimits^{\prime}(\eta )$

The effect of *M* on the *f(η )* and $\mathop{h}\nolimits^{\prime} (\eta )$ can be seen in Fig. 7(a) and (b) respectively. The Lorentz force generated by the applied magnetic field induces a decrease in the *f(η )* and $\mathop{h}\nolimits^{\prime} (\eta )$. The fluid is electrically conducting, so the magnetic field generates a drag force against the fluid flow. This resisting force grows as *M* increases, decreasing the momentum of the fluid and slowing its flow. Hence, the *f(η )* is found to be decreasing over the whole boundary layer area. In addition, the $\mathop{h}\nolimits^{\prime} (\eta )$ also decreases since the cross-flow is reduced by the *M* and the secondary flow characteristics are weakened. The magnetic field is like an absorbing force that absorbs kinetic energy from the fluid and transforms it into thermal energy, thereby decreasing the velocities of the fluid in both the axial and transverse directions.

### Temperature profile

**Fig. 8:**
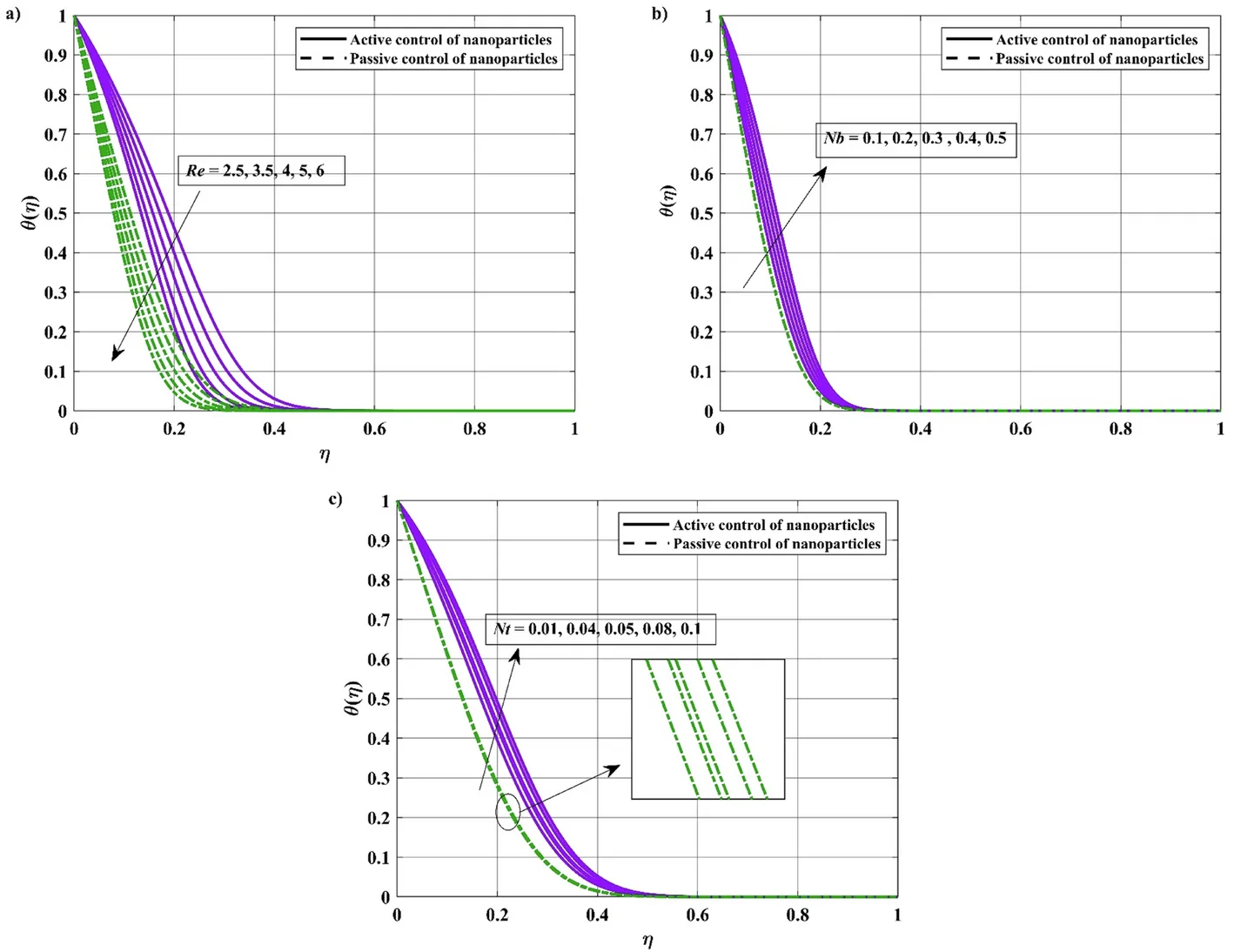
Impact of **a**) *Re***b**) *Nb***c**) *Nt* on $\theta (\eta )$

The temperature profile is shown decreasing with increasing *Re* in both cases of the active and passive control of nanoparticles in Fig. 8(a). This is attributed to the fact that the inertial forces have been increased and the flow of the fluid has been accelerated. This implies that the heat absorption by the fluid will be decreased, and therefore the temperature profile will be decreased. It is seen from Fig. 8(b) that the Brownian motion parameter (*Nb*) has an influence on the temperature profile ($\theta (\eta )$). The higher the *Nb* values, the higher the temperature profile under active control of nanoparticles. From a physical perspective, for a higher *Nb* value, the random movement and collision frequency of the nanoparticles in the fluid increase. These collisions produce more thermal energy, thus raising the temperature of the fluid and consequently, the thickness of the thermal boundary layer. Thus, the higher values of *Nb* increases the $\theta (\eta )$. In contrast, the effect of *Nb* on temperature is relatively small and is close to zero in the passive control case of nanoparticles, because of the passive mass flux boundary condition, which limits the transfer of nanoparticles near the wall surface. The temperature profile with the increase of the thermophoresis parameter (*Nt*) under the active nanoparticle control case is given in Fig. 8(c). The temperature difference physically aids the movement of nanoparticles from the hot wall to the cooler fluid regions (higher *Nt*). This movement enhances the thermal energy transfer and increases the thickness of the thermal boundary layer, resulting in greater temperature distribution. The temperature profile also rises as *Nt* increases for the passive nanoparticle control case. Even the thermophoretic force will push nanoparticles away from the heated surface, increasing heat transfer in the fluid. Therefore, under a passive flux boundary condition, the temperature profile increases.

### Nanoparticle volume fraction profile

**Fig. 9:**
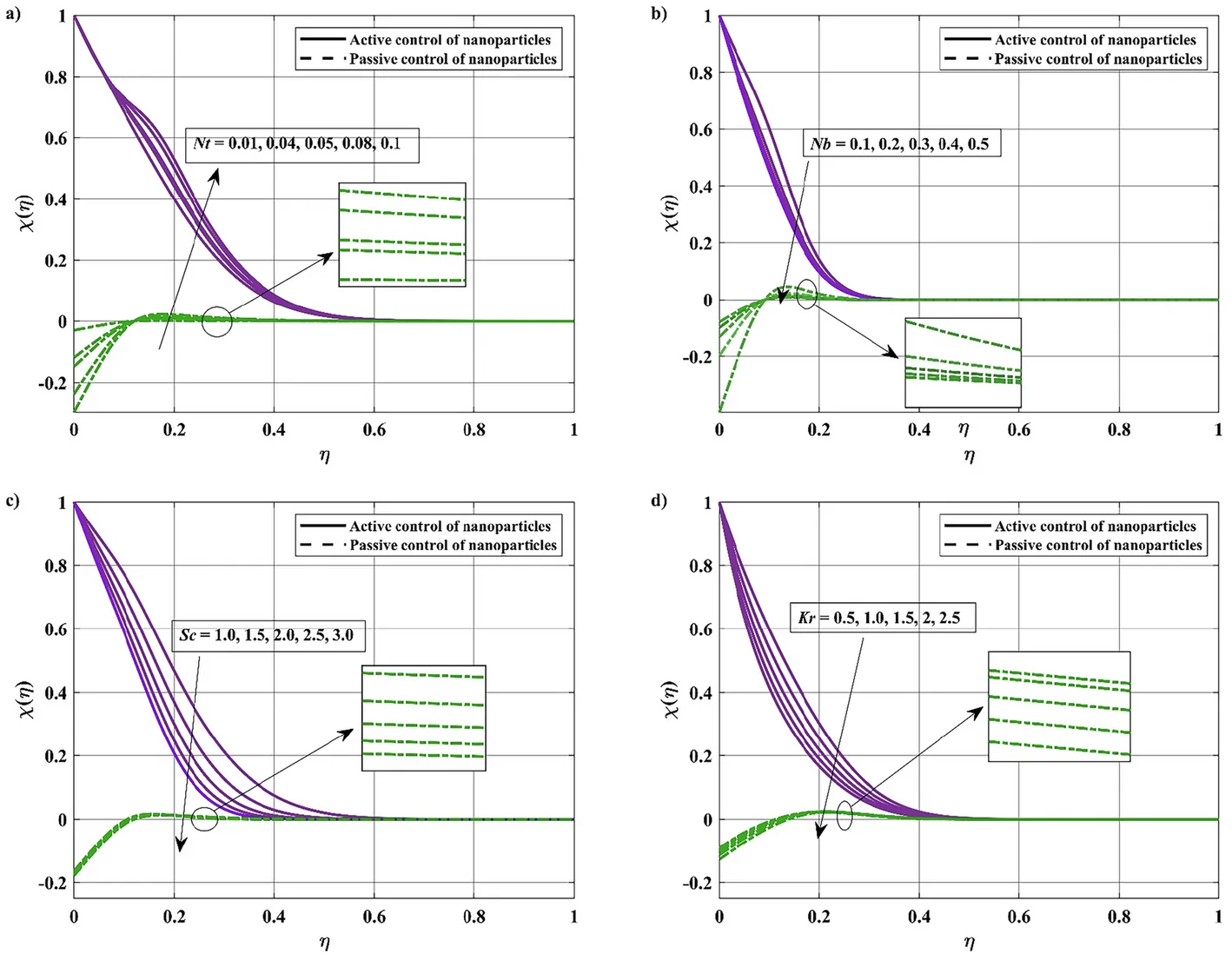
Impact of **a**) *Nt*, **b**) *Nb*, **c**) *Sc*, **d**) *Kr* on $\chi (\eta )$

The concentration profile for varied thermophoresis parameter *Nt* is plotted in Fig. 9(a) for both active and passive nanoparticle control cases, respectively, and it is found that the concentration profile increases with increasing *Nt*. This is due to the fact that high thermophoresis leads to a high concentration of nanoparticles in the boundary layer due to the migration of nanoparticles from the hot wall to the cold fluid area. In the active control, direct manipulation of the concentration of nanoparticles on the wall gives rise to high concentrations and hence increased particle transfer. In the passive control, both the Brownian force and thermophoresis lead to different behaviour of the concentration. Furthermore, due to the presence of activation energy, there is an increase in the diffusion of the species, leading to a thick concentration boundary layer and increased nanoparticle transfer in both cases. The concentration profile decreases with the increase of the Brownian motion parameter *Nb* in both cases of active and passive control of the nanoparticles, as shown in Fig. 9(b). When *Nb* increases, this will lead to an improvement in the random movement and diffusion of the nanoparticles within the fluid and they will diffuse very fast in the direction of the flow of the fluid away from the wall zone. Due to this effect there will be minimal deposition of the nanoparticles at the wall and there will be thinning of the concentration boundary layer. If the nanoparticles are actively controlled on the wall, the nanoparticles will experience enhanced Brownian diffusion, a process whereby the local concentration profile will be reduced. The passive scenario experiences greater Brownian motion, leading to better dispersion of the particles and a lower nanoparticle volume fraction in the boundary layer. Figure 9(c) shows that the increase in the Schmidt number (*Sc*) reduces the volume fraction profile of nanoparticles in both the cases of active and passive nanoparticle control. This behavior can be attributed to the fact that with an increase in *Sc*, the mass diffusivity decreases, making the penetration of the nanoparticles less into the fluid region. Therefore, the movement of nanoparticles becomes less effective, and there is a marked decrease in the concentration boundary layer. The trend of the profile remains decreasing with an increase in *Sc* despite the wall concentration conditions maintained by the active case, which keeps it at relatively high levels. In the passive case, since there is no control of the nanoparticles, the diffusive resistance increases, leading to a greater decrease in nanoparticle concentration distribution. In both active and passive nanoparticle control cases, the concentration profile decreases as the chemical reaction parameter *Kr* increases, as shown in Fig. 9(d). The higher the value of *Kr*, the greater the destructive chemical reaction rate in the fluid, and the more rapidly the nanoparticles are consumed in the fluid, the lower the concentration profile of the nanoparticles.

## Response surface methodology

RSM is a powerful statistical technique to model and study problems with several factors affecting one response of interest. The major objective of RSM is to construct an empirical model to depict input variables and output, which can be used to optimise and comprehend complex systems. It produces a polynomial equation that is used to approximate the functional relationship, which can be displayed on surfaces and in contour plots. These graphical tools show variable interactions and help to identify optimum solutions. RSM is useful in the chemical process industry, engineering, manufacturing, food technology and pharmaceuticals, where experiments are expensive, time-consuming, or resource-consuming. The present study aims to assess and enhance the heat transfer performance using RSM, in which several factors are taken into account that influence the thermal efficiency. The heat transfer rate is the dependent variable and the Reynolds number, the thermophoresis parameter and the Brownian motion parameter are independent variables within the specified ranges. Error percentages, P-values, F-values and model fit diagnostics provide evidence for the reliability and predictive power of the model. Sharma et al. [32] utilized RSM for statistical analysis and optimization of heat transfer properties of hybrid nanofluid flow over rotating conical-disk structures. However, Srilatha et al. [33] used RSM to optimise the model, yielding a high correlation with calculated results. Karthik et al. [34] used the Taguchi optimization based technique with analysis of variance (ANOVA) to explore the heat transfer in nanofluid flow.

### Central composite design

One of the most popular experimental designs for process development and optimisation in RSM is the central composite design (CCD). It aims to explore the factor-response correlation and to fit an adequate complete quadratic model. In a CCD, three sets of experimental runs are included: axial (or star) points, centre points and a factorial design. The centre points give information about experimental error and process stability and the factorial section shows information on linear and interaction effects. On the other hand, the axial points make it possible to estimate the response surface’s curvature. CCD can be visualised in a cube for three factors and a square for two factors, with the centre point at the origin, the star points along the coordinate axes to pick up the curvature and the corners representing the coded levels. CCD performs 27 experimental trials to capture the effects of each individual variable and the interaction between the variables. The procedure involves a three-level factorial design to study the main and interaction effects at high (+1) and low (−1) levels and centre points to determine the surface curvature. The ANOVA is used to check the statistical accuracy. The practical range of governing simulation parameters is shown in Table 5.

31 $$\begin{array}{l}-\theta^{\prime} (0)={\alpha }_{1}+A{\alpha }_{2}+B{\alpha }_{3}+C{\alpha }_{4}+A\ast A{\alpha }_{5}+B\ast B{\alpha }_{6}+C\ast C{\alpha }_{7}\\ \,\quad\qquad\quad+\,A\ast B{\alpha }_{8}+A\ast C{\alpha }_{9}+B\ast C{\alpha }_{10}.\end{array}$$

Here ${\alpha }_{i}(i=1-10)$ are the parameters of the whole quadratic model that the RSM generates.

**Table 5:** Range of the independent variables

Properties	Range
*Nt*	0.01 to 0.1
*Nb*	0.1 to 0.5
*Re*	2.5 to 6

### Comparison between active and passive cases

Active and passive controls are compared using regression statistics, predicted responses, error analysis and ANOVA results. The results obtained suggest that there are differences between the two control mechanisms in terms of thermal efficiency, response sensitivity and interactions among the parameters. The developed models for active and passive nanoparticle control cases are evaluated based on the following statistical indicators: P-values, F-values, coefficient of determination (R^2^) and standard error. In this study, the axial points are located at a unit distance from the center and the design region is square, which enhances the prediction accuracy and helps to find out the best operating conditions of the process. This approach can well reflect the effect of the key parameters and their interaction on the thermal performance parameters of the circular porous slider system, which can better realize accurate regression analysis.

ANOVA for the active and passive cases is employed to examine the adequacy and statistical reliability of the developed heat transfer prediction. The ANOVA results of the active and passive nanoparticle controls are presented in Tables 6 and 7, respectively. In Table 6, where the case for active control is considered, the proposed model is highly significant, having an F-value of 210.68. A P-value less than 0.05 is considered significant and the model accounts for 99.48% of the total variation. In linear terms, *Nb* is the most important factor, accounting for 75.92%, followed by *Re* (3.01%) and *Nt* (2.74%). The contribution of squared terms is 15.18%, mostly due to Nb* Nb (15.06%), while that of two-way interactions is 2.64%. Interactions of Nt* Nb and Nb* Re are statistically significant with a P-value of 0.001. This statistical approach evaluates the significance of the regression model by separating the total variation into different contributing sources, including linear terms, quadratic effects, interaction parameters and residual errors. Similarly, for the passive case in Table 7, the significance of the model is high, as it gives an F-value equal to 933.03. A P-value less than 0.05 is considered significant, explaining 99.88% of the total variation. The linear components explain 98.17% of the variation, where the major controlling component is *Re*, accounting for 98.07% of the variation, whereas *Nt* accounts for 0.11% and *Nb* has zero contribution, with a P-value of 1. The contribution of the square components is 1.71%, where most of the contribution is due to Re* Re, which is statistically significant. The contribution of the interaction effects is zero and this is statistically insignificant. The error contribution is 0.12%, which is less than that of the active case.

**Table 6:** Analysis of variance for active case

Source	DF	Adj SS	Seq SS	Contribution	Adj MS	F-Value	P-Value
Model	9	27.6387	27.6387	99.48%	3.071	210.68	0
Linear	3	22.6895	22.6895	81.66%	7.5632	518.86	0
*Nt*	1	0.7611	0.7611	2.74%	0.7611	52.22	0
*Nb*	1	21.0926	21.0926	75.92%	21.0926	1447.02	0
*Re*	1	0.8357	0.8357	3.01%	0.8357	57.33	0
Square	3	4.2164	4.2164	15.18%	1.4055	96.42	0
Nt* Nt	1	0.0085	0.0057	0.02%	0.0085	0.59	0.462
Nb* Nb	1	4.0751	4.1832	15.06%	4.0751	279.56	0
Re* Re	1	0.0276	0.0276	0.10%	0.0276	1.89	0.199
2-Way Interaction	3	0.7328	0.7328	2.64%	0.2443	16.76	0
Nt* Nb	1	0.3587	0.3587	1.29%	0.3587	24.61	0.001
Nt* Re	1	0.0147	0.0147	0.05%	0.0147	1.01	0.339
Nb* Re	1	0.3593	0.3593	1.29%	0.3593	24.65	0.001
Error	10	0.1458	0.1458	0.52%	0.0146		
Lack-of-Fit	5	0.1458	0.1458	0.52%	0.0292		
Pure Error	5	0	0	0.00%	0		
Total	19		27.7844	100.00%

**Table 7:** Analysis of variance for passive case

Source	DF	Adj SS	Seq SS	Contribution	Adj MS	F-Value	P-Value
Model	9	9.59293	9.59293	99.88%	1.06588	933.03	0
Linear	3	9.42902	9.42902	98.17%	3.14301	2751.25	0
*Nt*	1	0.01012	0.01012	0.11%	0.01012	8.85	0.014
*Nb*	1	0	0	0.00%	0	0	1
*Re*	1	9.4189	9.4189	98.07%	9.4189	8244.89	0
Square	3	0.16391	0.16391	1.71%	0.05464	47.83	0
Nt* Nt	1	0.00018	0.00235	0.02%	0.00018	0.15	0.703
Nb* Nb	1	0.00018	0.00285	0.03%	0.00018	0.16	0.696
Re* Re	1	0.15871	0.15871	1.65%	0.15871	138.93	0
2-Way Interaction	3	0	0	0.00%	0	0	1
Nt* Nb	1	0	0	0.00%	0	0	1
Nt* Re	1	0	0	0.00%	0	0	0.977
Nb* Re	1	0	0	0.00%	0	0	1
Error	10	0.01142	0.01142	0.12%	0.00114		
Lack-of-Fit	5	0.01142	0.01142	0.12%	0.00228		
Pure Error	5	0	0	0.00%	0		
Total	19		9.60436	100.00%			

The proposed regression equation for the active case is given as

32 $$\begin{array}{c}-\theta^{\prime} (0)=2.858-11.32Nt-12.911Nb+0.474Re+12.0Nt\ast Nt+13.294Nb\ast Nb\\ \quad\,\,\,\,-\,0.0143Re\ast Re+23.53Nt\ast Nb-0.544Nt\ast Re-0.606Nb\ast Re.\end{array}$$

The proposed regression equation for the passive case is given as

33 $$\begin{array}{c}-\theta^{\prime} (0)=2.2261-0.776Nt-0.054Nb+0.7661Re+1.72Nt\ast Nt+0.089Nb\ast Nb\\ \,-\,0.03427Re\ast Re+0.00Nt\ast Nb-0.004Nt\ast Re-0.0000Nb\ast Re.\end{array}$$

Results of statistical indicators like sums of squares, mean squares, degrees of freedom, F-values and p-values are analyzed to find out the influence and significance of each model component. The F-test is especially useful for the overall effectiveness of the regression equation developed and the p-values help to indicate the statistically significant parameters influencing the heat transfer rate. The complete assessment proves that the thermal model of the circular porous slider system is capable of accurate and strong prediction.

### Model validation

The ANOVA table evaluates the strength and accuracy of the regression model for both cases. ANOVA is an important statistical tool to check the adequacy of the proposed regression model and the reliability of the obtained response of heat transfer. The model developed is analysed at a 95% confidence interval and the significance level of 5% is maintained to ensure the credibility of the developed model. The p-values for each governing parameter and interaction terms are used to determine the contribution of each governing parameter. The parameters that have a p-value below 0.05 are considered statistically significant in the description of the thermal behaviour of the system. All model terms were significant (except Nt* Nt and Nt* Re for the active case and *Nb*, Nt* Nt, Nb* Nb,Nt* Nb, Nb* Re and Nt* Re for the passive case, with p-values greater than the significance level). Hence, the non-significant terms were omitted from the regression formulation, which resulted in a better and more statistically stable prediction model of the heat transfer characteristics of the circular porous slider system. After the validation, the regression equations are as follows:

Active case:

34 $$\begin{array}{c}-\theta^{\prime} (0)=2.858-11.32Nt-12.911Nb+0.474Re+13.294Nb\ast Nb\\ \quad\,\,\,\,+\,23.53Nt\ast Nb-0.606Nb\ast Re.\end{array}$$

Passive case:

35 $$-\theta^{\prime} (0)=2.2261-0.776Nt+0.7661Re-0.03427Re\ast Re.$$

### Summary of the models

Tables 8 and 9 show a value of 99.48% for ${R}^{2}$ and an adjusted ${R}^{2}$ of 99.00% for the active case, and for the passive instance, the value of ${R}^{2}$ is 99.88% and an adjusted ${R}^{2}$ of 99.77%, along with a thorough description of the model. ANOVA was used to determine the model’s reliability and the results are shown. For each scenario, the standard error of regression is 0.120733 and 0.0337993. The passive case has a lower standard error than the active case, according to the regression analysis. The passive control exhibits a lower standard error of regression, indicating that the data are more closely clustered since the response predicted by this case has a smaller variance around the data points. Both the statistical fit and prediction consistency of the passive control model are better than those of the active control situation. Regression error is greater in the active control arrangement due to the comparatively larger difference between predicted and actual values. As a result, from the perspective of statistical modelling, the passive control mechanism provides a more stable and trustworthy representation of the transfer phenomena that are the subject of the current study. The validity of the built models may be inferred from error analysis of the active and passive instances. The findings match the model’s predictions and the prediction error is tiny, as shown by the small value of S and larger ${R}^{2}$. Thus, the error analysis validates the present research by demonstrating that the employment of active and passive models is sufficient and predictable. PRESS statistics values for the active and passive cases are 1.11872 and 0.08674, respectively. Smaller PRESS values indicate better predictive performance.

**Table 8:** Model summary for the active case

S	${R}^{2}$	${R}^{2}$ (adj)	PRESS	${R}^{2}$ (pred)
0.120733	99.48%	99.00%	1.11872	95.97%

**Table 9:** Model summary for the passive case

S	${R}^{2}$	${R}^{2}$ (adj)	PRESS	${R}^{2}$ (pred)
0.0337993	99.88%	99.77%	0.08674	99.10%

### Graphical results for the active-passive case

**Fig. 10:**
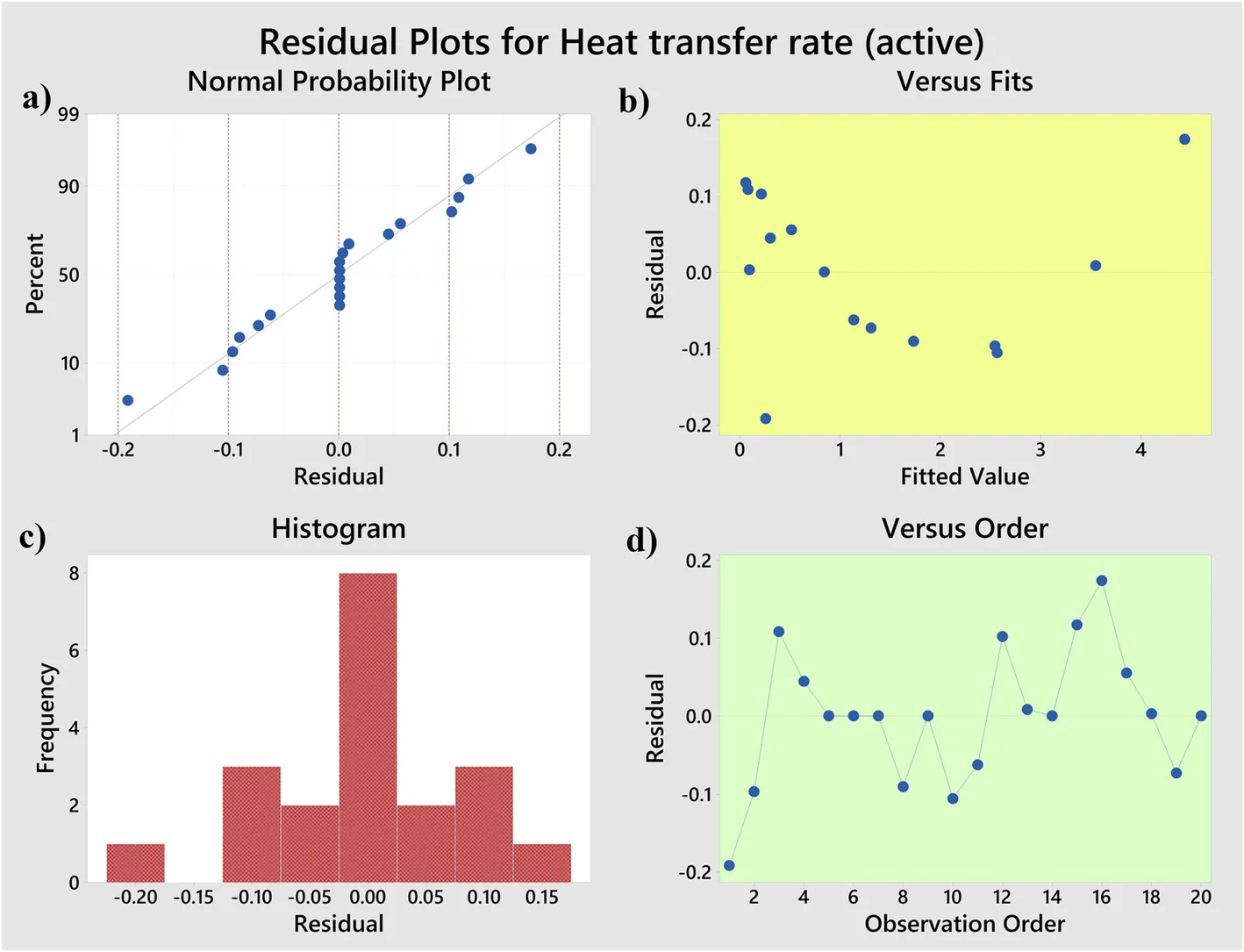
Residual plot for the heat transfer rate for the active case **a)** Normal probability plot, **b)** Versus fits, **c)** Histogram, and **d)** Versus order

**Fig. 11:**
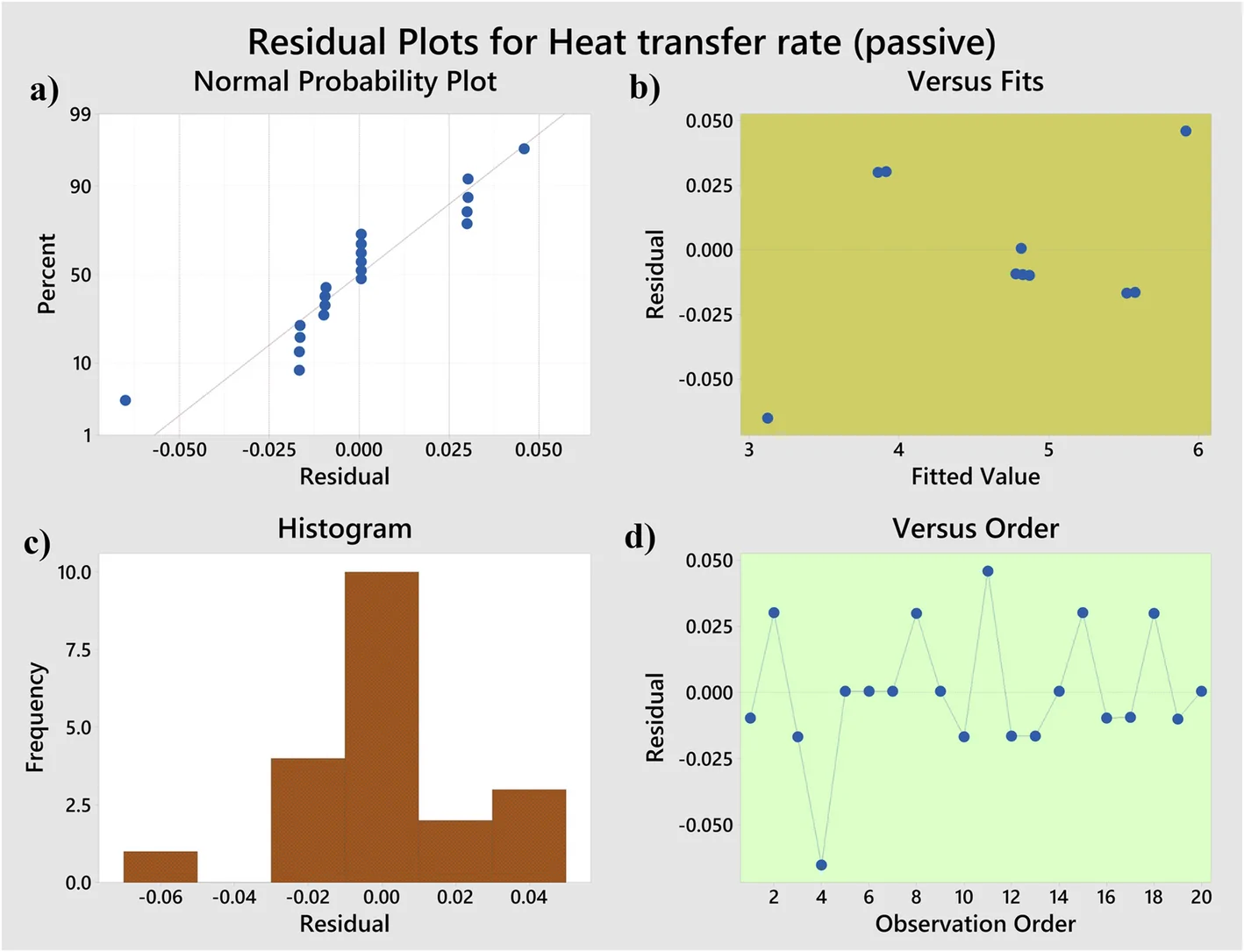
Residual plot for the heat transfer rate for the passive case **a)** Normal probability plot, **b)** Versus fits, **c)** Histogram, and **d)** Versus order

**Fig. 12:**
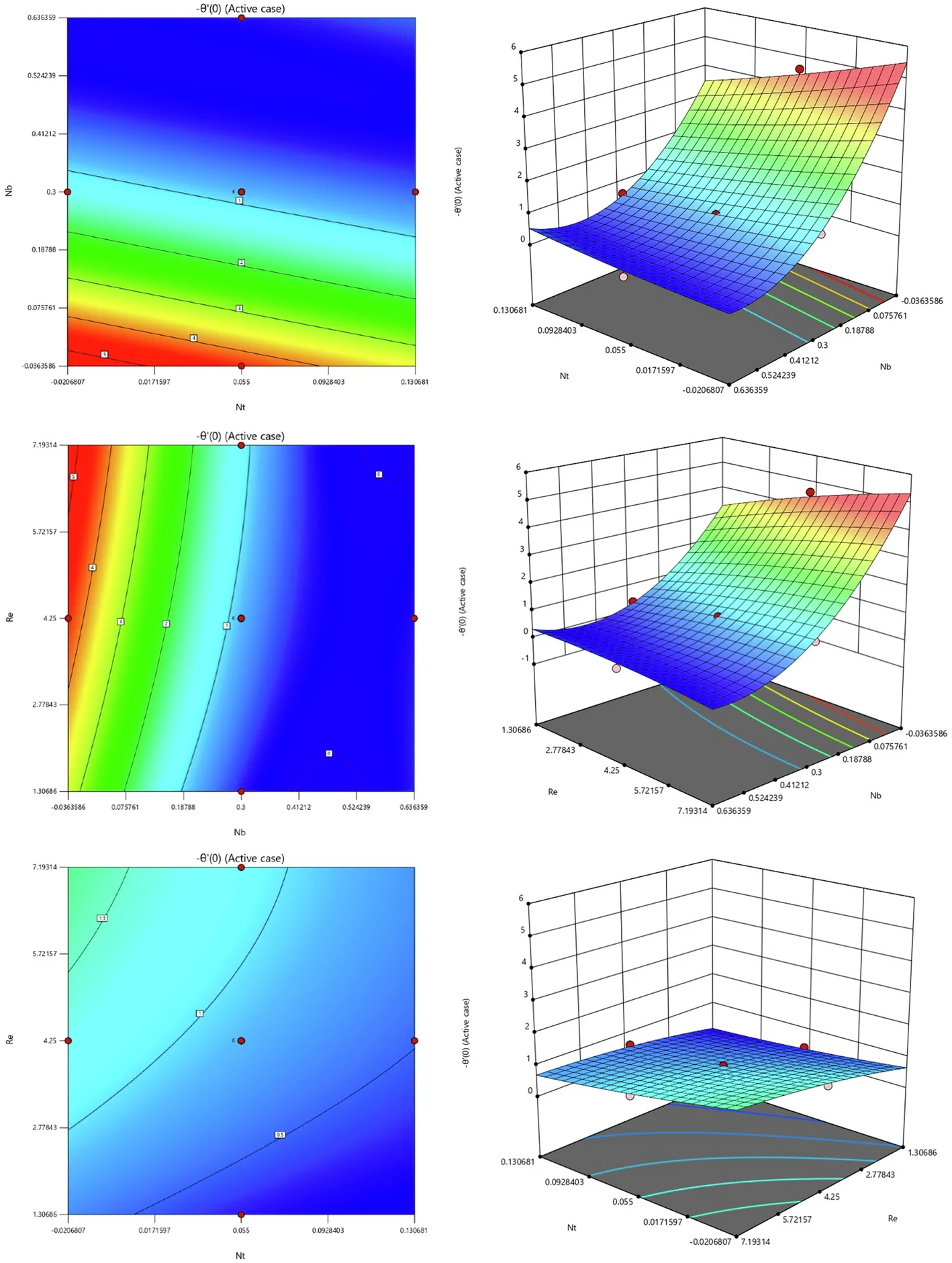
Contour and surface plot for the active case

**Fig. 13:**
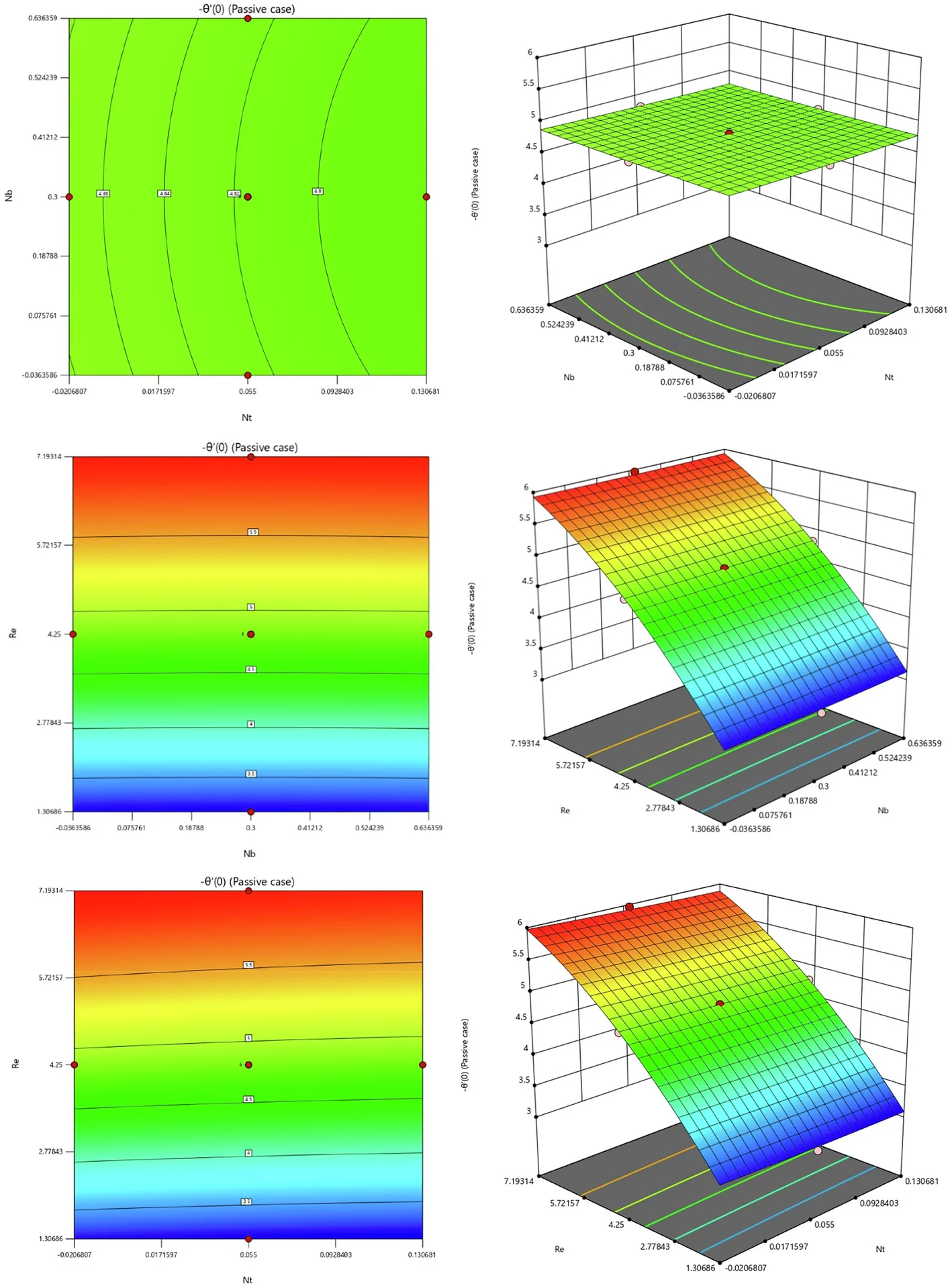
Contour and surface plot for the passive case

The normal probability plot is shown in Fig. 10(a) for the active case, where the residual points are plotted to lie closely along the straight line of reference. It signifies that the residual points are normally distributed except for very minor deviations at the tail ends. The residual versus fitted values plot is illustrated in Fig. 10(b). Here, the residual points are randomly distributed around the zero line without showing any pattern. This implies that there is no autocorrelation or heteroscedasticity among the residuals. The histogram of residuals is illustrated in Fig. 10(c). The residual points in this plot are almost evenly distributed around the zero line, implying unbiasedness in the residual distribution. The residuals versus observation order plot is illustrated in Fig. 10(d). The residual points in this plot are randomly distributed around the zero line without any trend, implying that the residuals are independent of each other. The normal probability plot for the passive case is shown in Fig. 11(a). It shows that the residual points lie near the reference straight line, suggesting that the residuals are normally distributed with slight deviation from normality at the extreme ends. The residuals versus fitted values plot, shown in Fig. 11(b), shows that the residuals lie randomly along the zero-reference line without following any definite pattern, thus validating the assumption of constant variance and the adequacy of the developed RSM model. In the histogram of residuals in Fig. 11(c), the residuals are almost symmetrically distributed about zero, showing that the errors in prediction are minimal. Figure 11(d) depicts the residuals versus observation order plot, where the residuals show random fluctuations about the zero-reference line without any increasing or decreasing trend, suggesting the independence of residuals. Therefore, the residual analysis validates the statistical reliability, accuracy and applicability of the passive heat transfer rate model for prediction and optimization. In comparison, the passive heat transfer rate model demonstrates superior statistical performance compared to the active heat transfer rate model, since the deviations of the residuals from zero are smaller and hence the spread is also lower. The diagnostic tests on the residuals have confirmed that the two RSM models developed for active and passive heat transfer rates are statistically valid and accurate. Whereas Figs. 12 and 13 describe the contour and surface plots for the active and passive cases, respectively.

## Artificial neural network

An ANN is a computational intelligence technique based on the biological structure of the human brain, developed to learn and model complex nonlinear relationships between the input and output variables. It is made up of interconnected neurons arranged in input, hidden and output layers, with the input layer taking in the data, hidden layers performing mathematical calculations with the neurons using weighted connections and activation functions and the output layer producing the final prediction. As the network is trained, it updates its weights and biases to reduce the errors of its predictions and increase its accuracy. ANN is well known for its powerful learning ability, adaptability and predictive ability in complex engineering and scientific problems. Due to its excellent nonlinearity treatment characteristics, ANN has been widely used in the areas of optimisation, prediction, classification, image processing, fluid mechanics and heat transfer analysis to predict physical quantities like velocity, temperature, concentration profile, skin friction and heat transfer rate, with low computation requirements and high accuracy as compared to traditional numerical methods. Sharma et al. [35] applied the LM-ANN model for predicting hybrid nanofluid flow, obtaining satisfactory results with minimum errors and good correlation with numerical solutions. Banakar et al. [36] used ANN to predict the thermal profile using linear, nonlinear and quadratic radiation models, indicating the efficiency of ANN in thermal-flow prediction. Kattimani et al. [37] explored an ANN-Taguchi framework for prediction and optimization of heat transfer, showing its robustness for heat and mass transfer systems. Kumar et al. [38] utilized LM-ANN model for predicting for optimization of heat transfer properties of nanofluid flow between disks.

### Training strategy of levenberg-marquardt artificial neural network

**Fig. 14:**
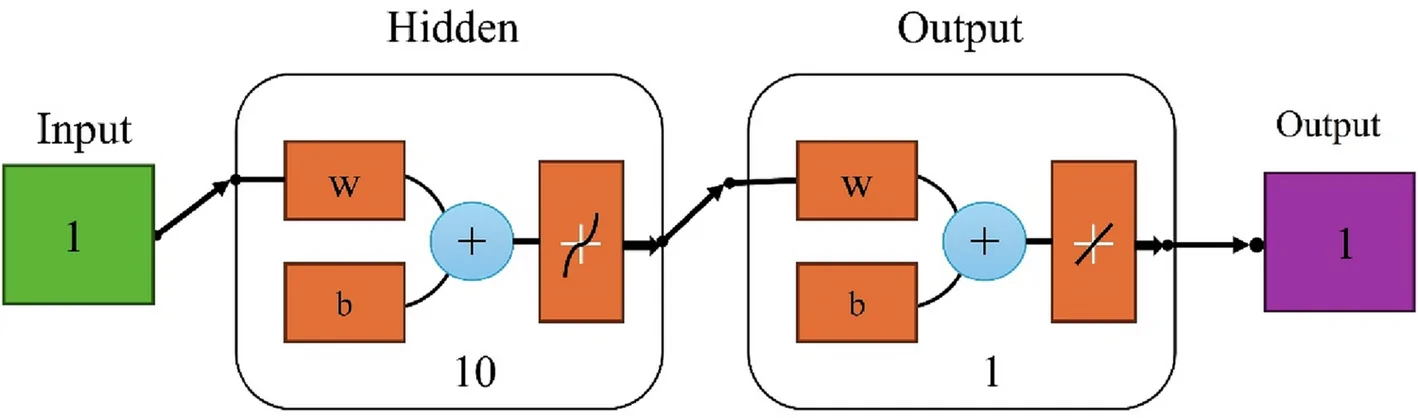
The training strategy of the LM-ANN

The schematic model and computation mechanism of the ANN model created in this work are shown in Fig. 14. The training, validation and testing data sets are generated using the RKF-45 approach. The results of these numbers are then estimated by an ANN model with the LM method. In the context of single-output networks and problems pertaining to second-order training, this strategy was favoured. The LM approach is more accurate and computationally efficient. The model is implemented in the MATLAB neural network toolbox, which allows networks to be systematically designed, trained and tested. The data will then be split into three sets: 70% for training, 15% for validation and 15% for testing, so that there is a similar amount of data to evaluate the model’s performance on new data. The choice of network parameters, such as the number of hidden neurons, greatly influences the prediction ability of the ANN model. This research will be conducted to characterize the structure of the network and to find the best topology. It is demonstrated that a network with 10 hidden neurons will be the most economical and accurate when predicting. The model performance is assessed using quantitative measures of error, such as the mean squared error (MSE), the absolute error (AE) and the discrepancy between the expected and goal performance.

Considering the efficient hybrid optimisation capability of the LM-ANN approach, which combines the merits of the gradient descent method and Gauss-Newton method, faster convergence and more accurate prediction are achieved in the present study. The reference data is created over the parameter space using the numerical technique RKF-45. Careful tuning of the learning rate, hidden neuron number, regularisation and an appropriate division of the dataset into training and test sets are used to minimise prediction errors, thereby avoiding overfitting or underfitting. The performance and reliability of the trained model are analysed via various statistical and graphical methods such as the MSE, regression plot, fitness curve and fault histogram. Progressive decrease of MSE during the training process is a sign of learning, while tight clustering of the errors around zero and a good match between the predicted and the target value mean that the model is accurate, stable and has high generalisation power for the nonlinear engineering problem.

### Graphical overview

**Fig. 15:**
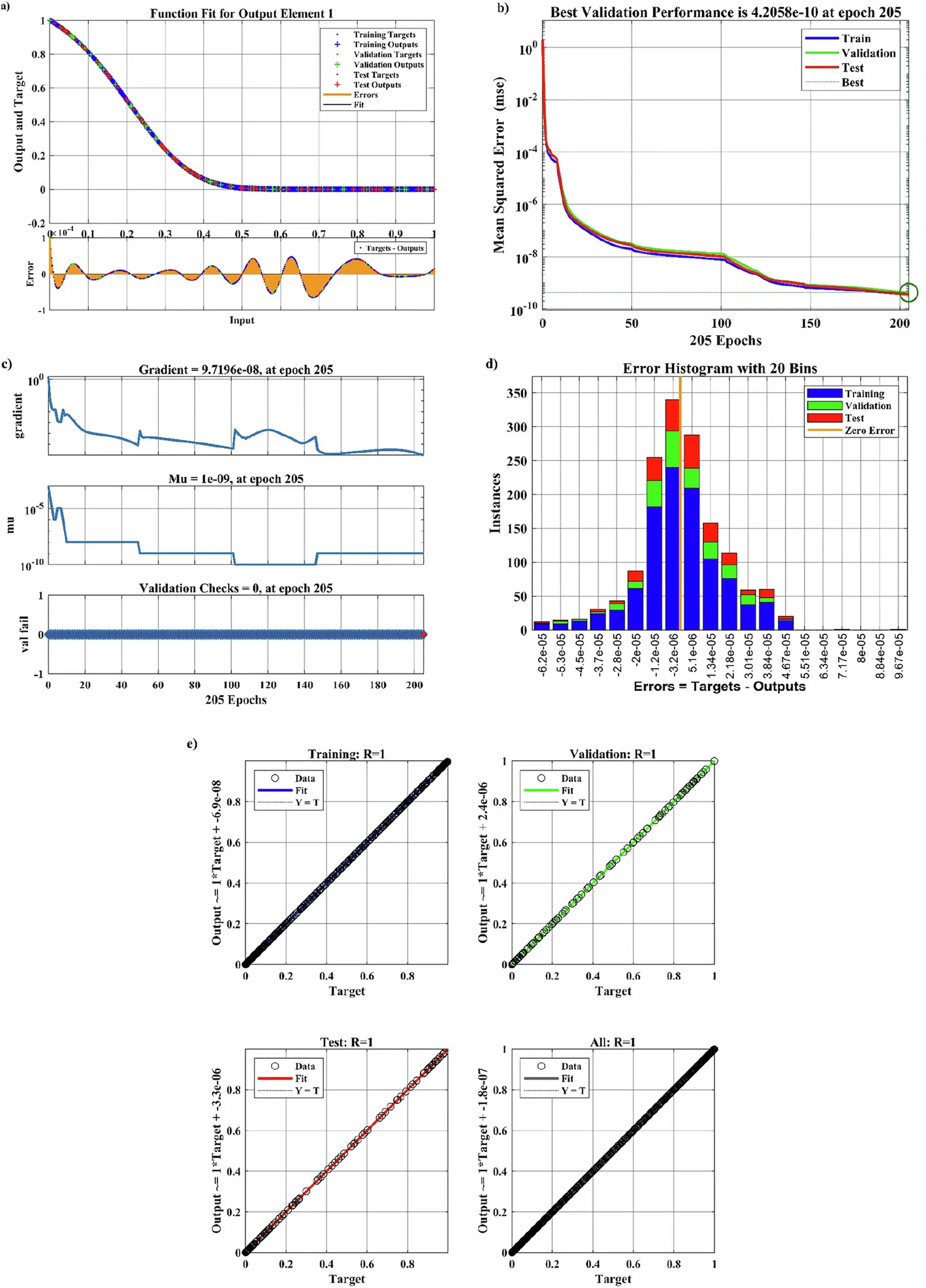
LM-ANN for the temperature profile with varying *Re* for the active case **a)** Fit curve, **b)** Performance, **c)** Training state, **d)** Error histogram, and **e)** Reggresion

The excellent predictive ability and convergence behaviour of the developed LM-ANN model can be observed in Fig. 15. Function fitting between the target output and the predicted output is presented as shown in Fig. 15(a), which almost completely overlaps both curves, meaning that the prediction accuracy is very high and the residual errors are very small. The lower error plot also demonstrates that the differences between the target output and the network outputs are negligible in the whole computational domain. The validation performance curve is plotted in terms of MSE in Fig. 15(b) and the best validation performance $4.2058{e}^{-10}$ is achieved at epoch 205, which means that the network has reached an excellent level of learning efficiency and minimal prediction error. The training, validation and testing curves are in close agreement, indicating that the system is converging without overfitting, as can be seen in Fig. 15(c), where the training state parameters such as gradient, Mu and validation checks are shown. The gradient value reduces drastically to $9.7196{e}^{-08}$, indicating that the optimisation has reached a good minimum value. At the same time, the small Mu of the model shows good optimisation behaviour to the LM and the model’s number of validation failures during training is zero, which shows that the generalisation ability of the model is good. The error histogram is shown in Fig. 15(d), in which the errors in prediction are concentrated around the origin, indicating very low deviation between the actual and predicted values and thus high reliability of the network. Figure 15(e) highlights the regression plots. It supports the capability of the ANN, as the correlation coefficient *R=1* shows that the outputs of the ANN have almost a linear relationship with the outputs of the target data set for the training, validation, testing and overall datasets. Overall, the graphical results validate that the developed LM-ANN model successfully reaches very good convergence and stability, as well as accuracy and generalisation capability for the case of active Reynolds number.

**Fig. 16:**
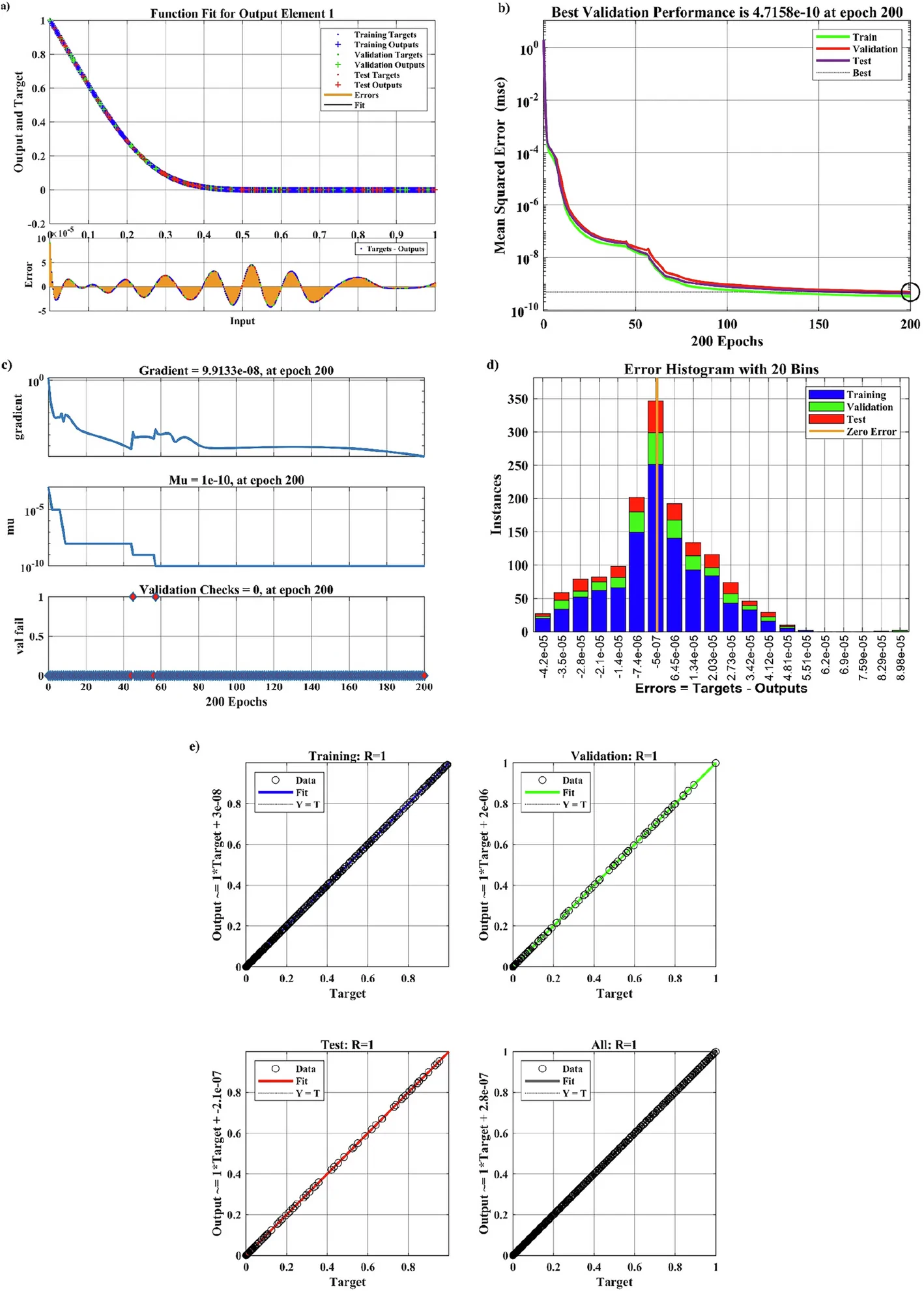
LM-ANN for temperature profile with varying *Re* for the passive case **a)** Fit curve, **b)** Performance, **c)** Training state, **d)** Error histogram, and **e)** Reggresion

The graphical results of the passive *Re* case showed that excellent learning capability, convergence and prediction of the developed LM-ANN model took place. As shown in Fig. 16(a), the predicted outputs are almost entirely in agreement with the target data in the training, validation and testing phases, which illustrates the high accuracy of function fitting and the good approximation of the nonlinear flow behavior. The error curve associated with it stays very small throughout the computational domain and shows that there is not much variation between the actual and the predicted output. As seen in Fig. 16(b), the MSE error curve, the validation error steadily decreases and reaches the minimum value $4.7158{e}^{-10}$ at epoch 200. Because the training, validation and testing curves are so close together, it shows good learning and stable convergence, with no evidence of overfitting. The training state behaviour is presented in Fig. 16(c) and it is observed that the gradient value was very small $9.9133{e}^{-08}$ and the Mu was small ${e}^{-10}$, which confirms the achievement of the error minimum region and efficient optimisation. There are a few changes in the validation checks, which continue to be near constant, indicating stable generalisation behaviour. Figure 16(d) presents the error histogram, with most errors being very close to zero, indicating that the predictions are very accurate and there is little variation in the outputs. The regression plots as seen in Fig. 16(e), show almost perfect fits of the predicted and target outputs for training, validation, testing and overall datasets, respectively, with the correlation coefficients (*R=1*) signifying the robustness, reliability and excellent predictive capability of the ANN model for the passive Reynolds number case.

**Fig. 17:**
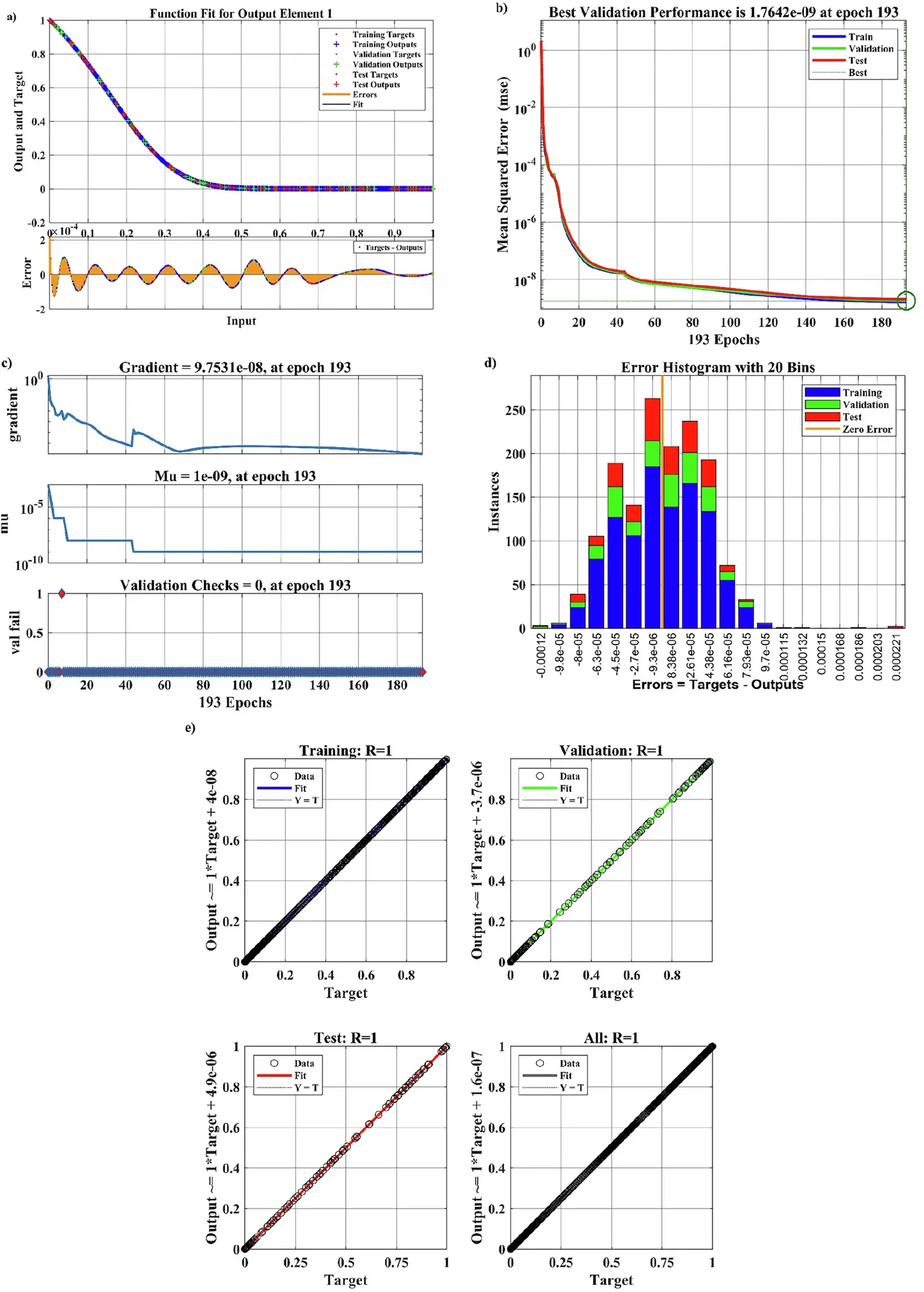
LM-ANN for temperature profile with varying *Nt* for the active case **a)** Fit curve, **b)** Performance, **c)** Training state, **d)** Error histogram, and **e)** Reggresion

The ANN graphical results for the *Nt* of the active case show very good convergence behavior, prediction accuracy and stability of the developed LM-ANN model. By comparing the predicted outputs with the target values in Fig. 17(a), it can be seen that the predicted results are in close agreement with the target values for the training, validation and testing sets, indicating a very good fit of the functions and good approximation of the nonlinear thermophoretic behaviour. The related error profile is still very small, showing only small oscillatory deviations, which confirms that there is not really any difference between the actual and predicted outputs. The error pattern remains very minimal with only slight oscillations, which indicates there is no substantial difference between the theoretical and actual outputs. Figure 17(b) shows the validation performance curve, showing the decrease in MSE with the increase in epochs, till epoch 193, at which the best validation performance is achieved, which is $1.7642{e}^{-09}$. Notice how close the training, validation and testing curves are; it shows stable learning and good generalisation with not much overfitting occurring. The training state performance is shown in Fig. 17(c) with the gradient closing in on $9.7531{e}^{-08}$, which shows that the optimisation process is converging to the region of minimum error desirable. The very small Mu value ${e}^{-09}$ further validates the stable and efficient convergence properties of the LM optimisation algorithm and the validation checks are almost the same, indicating consistent predictive capability during optimisation training. The error histogram given in Fig. 17(d) shows the distribution of errors; most errors are within a small range around zero, suggesting a high accuracy of prediction and strong reliability of the model. The regression plot can be seen in Fig. 17(e). The plots also support the performance of the ANN, showing a near-perfect agreement between the outputs of the target and the predicted values for training, validation, testing and overall datasets, with correlation coefficients *(R=1)* respectively. In conclusion, the graphical results obtained agree well with the results obtained from the LM-ANN model, showing that the LM-ANN model is very useful in predicting the case of active thermophoresis with highly accurate, stable and reliable results.

**Fig. 18:**
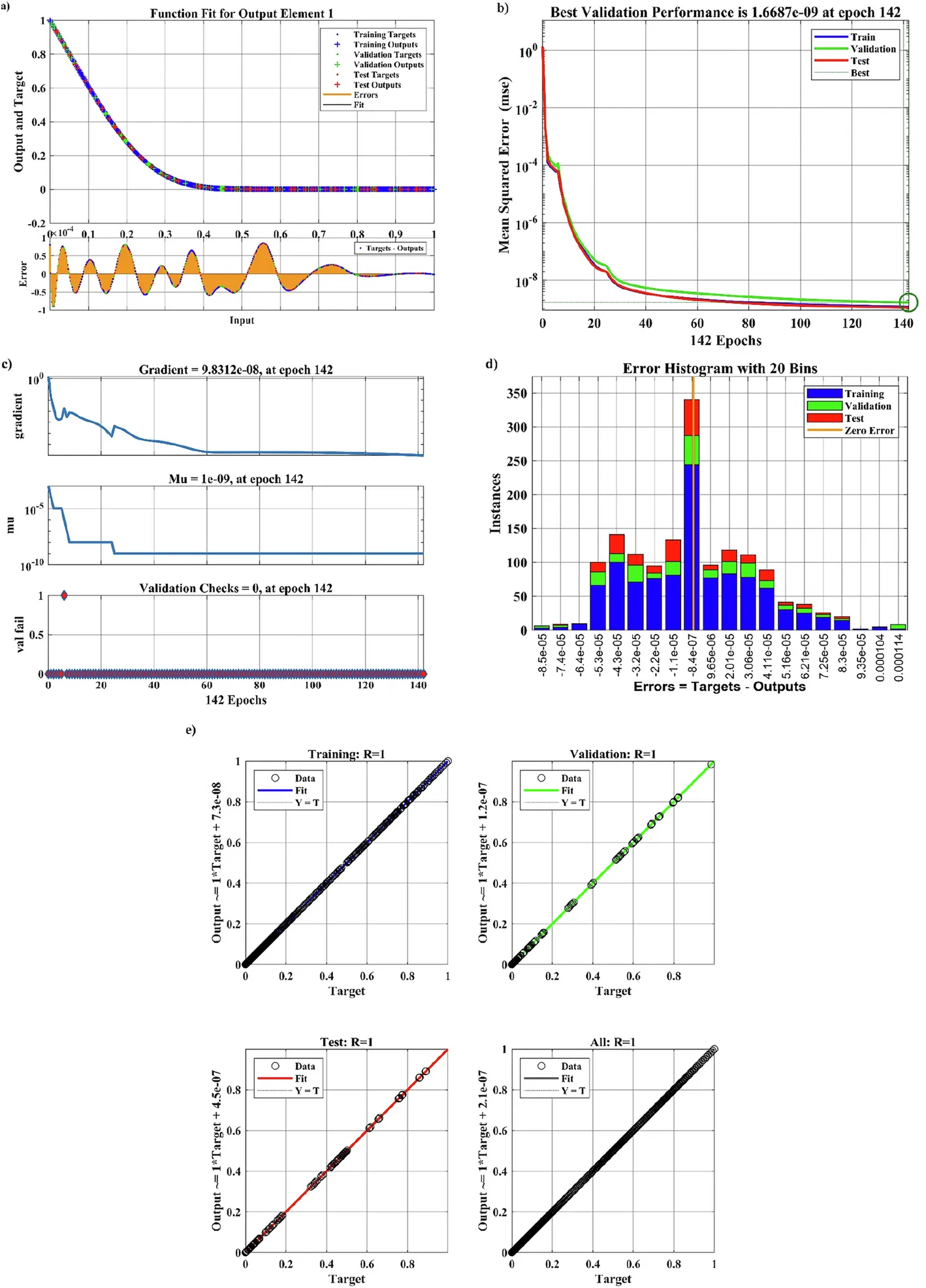
LM-ANN for temperature profile with varying *Nt* for the passive case **a)** Fit curve, **b)** Performance, **c)** Training state, **d)** Error histogram, and **e)** Reggresion

The performance plots of the ANN for the passive case for *Nt* show good prediction accuracy, along with good convergence stability and good learning capability of the developed LM-ANN model. The outputs are very similar to the target values for the training, validation and testing sets, as seen in Fig. 18(a), which shows that a very good function fitting and a good approximation of the nonlinear thermophoretic behaviour are obtained. The error distribution under the fitting curve is still very small and with minimal oscillations, indicating there is very little variation between the actual and predicted output. The validation performance curve of Fig. 18(b) shows that the MSE of the model decreases gradually with the number of epochs and reaches the minimum validation performance $1,6687{e}^{-09}$ at epoch *142*. All three curves are close to each other, suggesting well-balanced learning and there is no obvious overfitting or underfitting. Figure 18(c) shows the behaviour during the training process and shows that the optimisation process is close to the minimum error region with the gradient becoming $9.8312{e}^{-08}$. The *μ * value is quite low ${e}^{-09}$, demonstrating the stable convergence property of the Levenberg–Marquardt optimisation algorithm and the validation checks stayed almost unchanged throughout the training process, thus indicating stable prediction performance. The error histogram is presented in Fig. 18(d) and it shows that most of the prediction errors are very close to zero, which represents high accuracy and reliability of the model and low variation in the output. The regression plots in Fig. 18(e) also show that the ANN model is well-suited, with *R=1* on both the training, validation and testing sets and the overall set. It indicates that there was almost a perfect correlation between the target and the output of the ANN model. Overall, the graphical results demonstrate the high accuracy and stability of the passive thermophoresis parameter case predictions by the LM-ANN model and that they are reliable.

### Comparison between LM-ANN and numerical technique

**Fig. 19:**
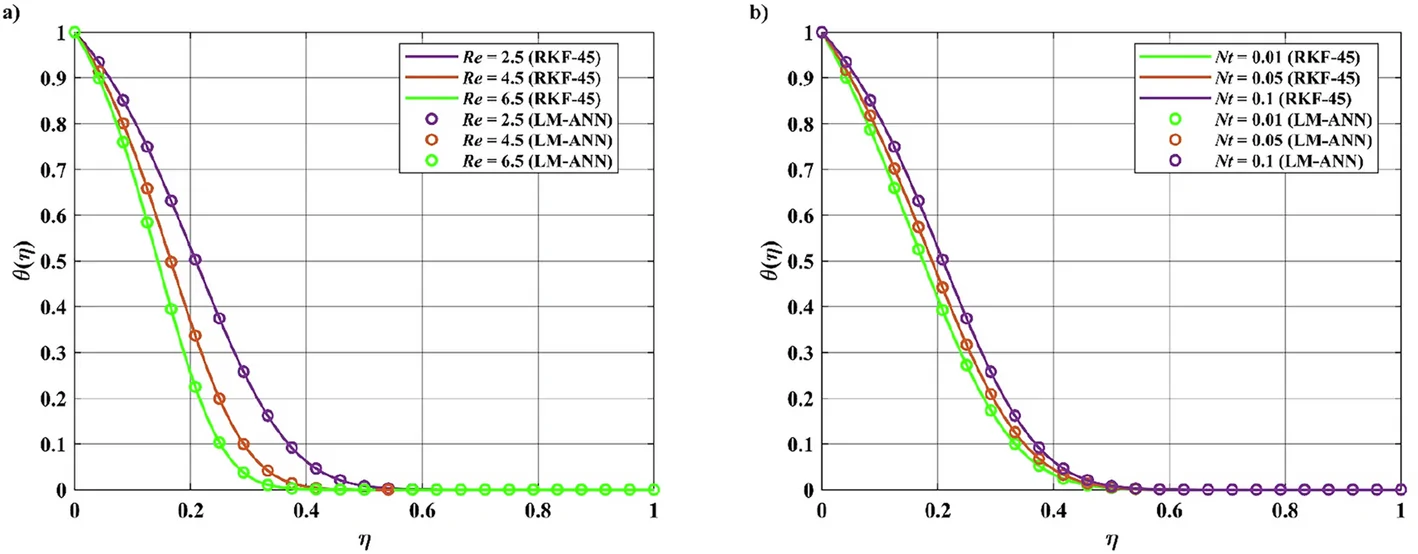
Comparison of LM-ANN and RKF-45 for various **a)** *Re* and **b)** *Nt* for the active case

The comparison of the LM-ANN and RKF-45 solutions for the active case shows that the developed ANN model has excellent predictive capability and reliability, as seen in Fig. 19. The LM-ANN predictions are in excellent agreement with the RKF-45 numerical solutions, with very little prediction error, both in *Re* and *Nt* cases over the entire computational domain. In the *Re* case the $\theta (\eta )$ is decreasing with increasing *Re*, indicating thinning of the thermal boundary layer with enhanced inertial effects and fluid flow. Likewise, in the thermophoresis parameter case, the increase in the temperature profile with increasing *Nt* reveals that stronger thermophoresis forces help to thicken the thermal boundary layer and improve the thermal energy transfer in the fluid. The agreement between the near-coincidence of the circular LM-ANN data points and the continuous RKF-45 curves validates the developed LM-ANN model, which is able to capture the nonlinear thermal behaviour of both parameters. The slight difference between the two models confirms the high accuracy, good convergence and good generalization ability of the ANN framework in the active transport mechanism.

**Fig. 20:**
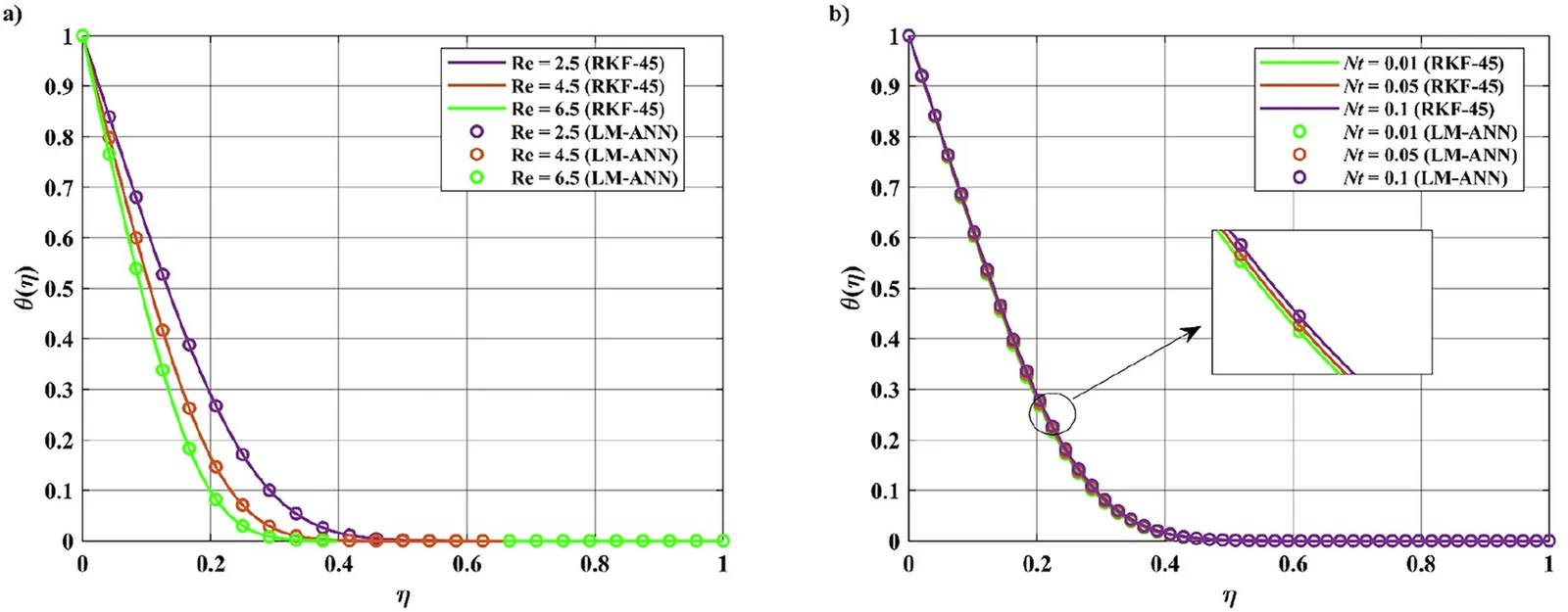
Comparison of LM-ANN and RKF-45 for various **a)** *Re* and **b)** *Nt* for the passive case

The comparison plots for the passive case in Fig. 20 between LM-ANN and RKF-45 solutions further confirm the excellent predictive accuracy and strength of the developed ANN model. In both *Re* and *Nt* cases, the LM-ANN predictions are in good agreement with numerical solutions (RKF-45) with little deviation over the entire computational domain. The Reynolds number case shows that with increasing *Re*, the $\theta (\eta )$ decreases, which means that the thickness of the thermal boundary layer decreases because of the increase in the inertial forces and the improved flow of the fluid as *Re* increases. For the thermophoresis parameter case, the temperature profile increases slightly with an increase in *Nt* while thermal diffusion increases in the presence of thermophoresis, which increases the thickness of the thermal boundary layer. The large inset further demonstrates the high level of precision and convergence that is achieved with this ANN framework, even in very small profile changes, thereby showing the high level of accuracy and convergence achieved by the LM-ANN data points and RKF-45 curves. Considering the very low error obtained between the two methods, it confirms that the developed LM-ANN model can capture the nonlinear thermal behavior of the passive transport mechanism in an excellent manner with both reliability and computational efficiency.

### Error analysis for active and passive cases

**Fig. 21:**
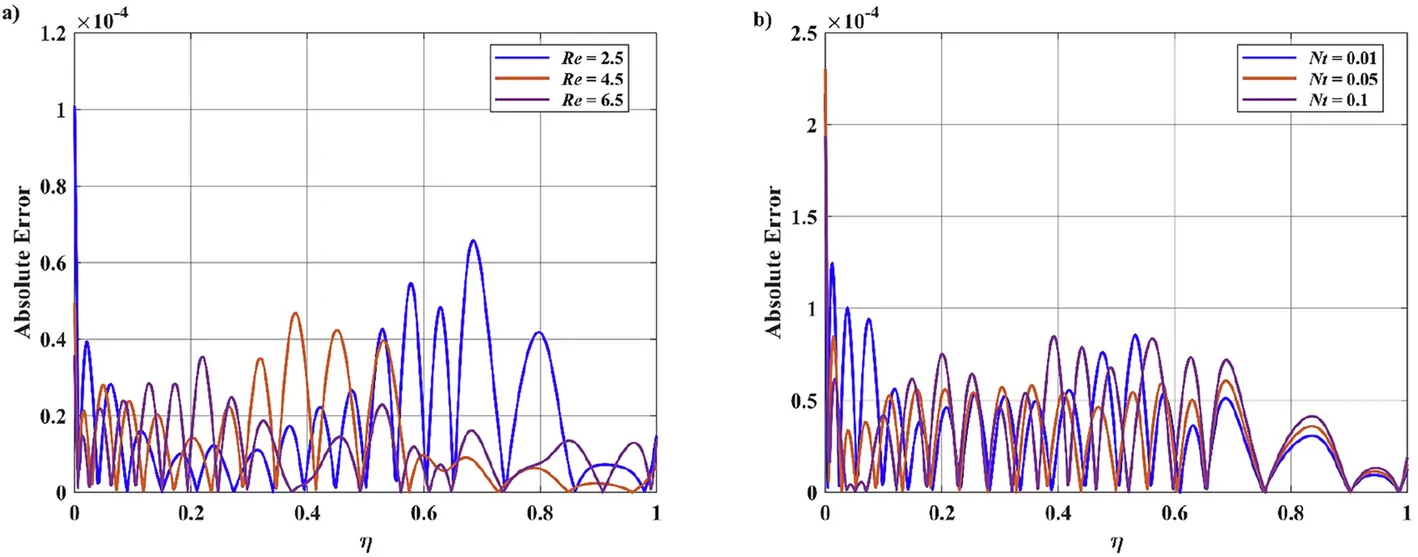
$\theta (\eta )$ for various **a)** *Re* and **b)** *Nt* with the absolute error for the active case

The AE analysis plots for the *Re* and the *Nt* in the active case confirm the high accuracy and good predictive power of the model developed by LM-ANN as seen in Fig. 21. In both cases, the AE values are very small at all points of the computational domain and vary mainly around the order of ${10}^{-4}$, which shows that the deviation between the solutions given by the LM-ANN and the RKF-45 is negligible, as can be seen in Fig. 21(a) and (b). The error profiles for the Reynolds number case show slight oscillatory variations as a function of the *η * value, with somewhat larger errors when the *Re* is lower. The errors are very low, which shows good approximation behaviour and strong approximation of the ANN model. Likewise, in the thermophoresis parameter case, the AEs are small and evenly scattered over the domain and have a consistent level of predictive performance for various *Nt* values. Slight variations in the error curves are caused by the extremely nonlinear coupling between thermal and concentration fields for the active transport mechanisms. However, the magnitudes of the overall error are extremely small, which proves that the developed LM-ANN can well reproduce the nonlinear physical behaviour of both *Re* and *Nt* parameters with high accuracy, reliability and computational efficiency.

**Fig. 22:**
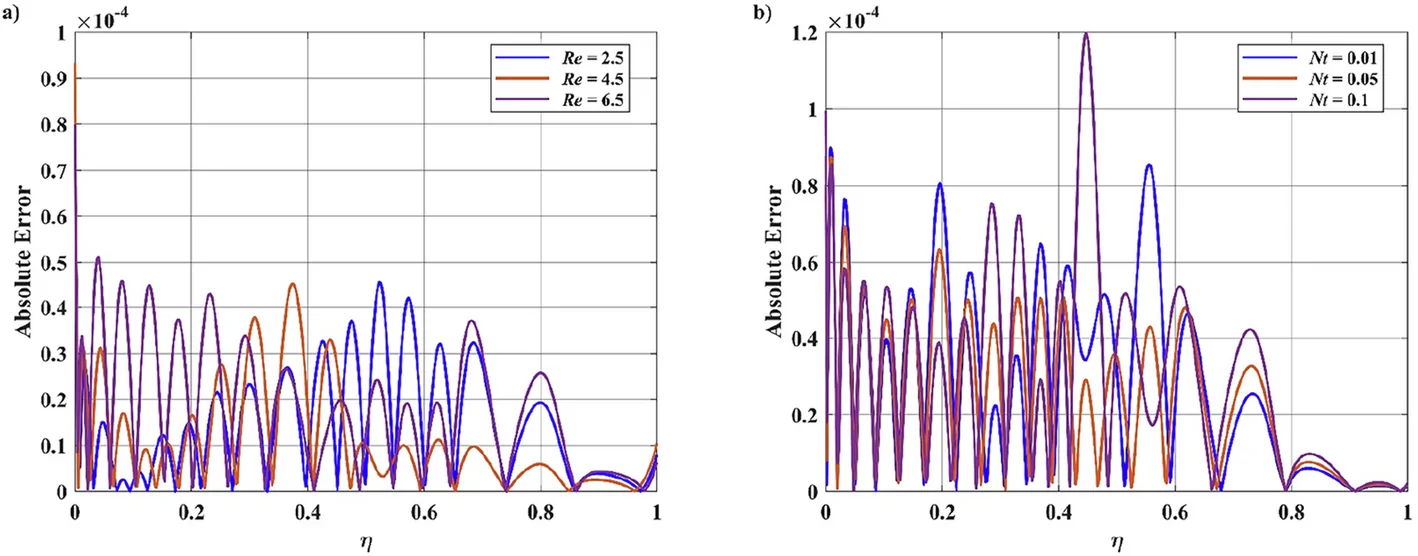
$\theta (\eta )$ for various **a)** *Re* and **b)** *Nt* with the absolute error for the passive case

The error analysis plots for the *Re* and *Nt* in the passive case are shown in Fig. 22(a) and (b) respectively. It shows the predictive accuracy and convergence behavior of the developed LM-ANN model. The AEs for both cases are very small and of the order of ${10}^{-04}$, which indicates a very small difference between the AEs of LM-ANN and the numerical solution RKF-45. The Reynolds number case yields small oscillatory variations in the error profiles throughout the computational domain; the oscillatory variation is slightly higher for the larger Reynolds number values because of the increased inertial effects and flow interactions that are nonlinear. The overall error magnitude is, however, still very small, which shows a stable approximation capability and accurate prediction of the thermal behavior. Likewise, in the case of the thermophoresis parameter, the AE curves show small oscillatory fluctuations with slightly higher peaks for higher *Nt* values, due to the stronger direct coupling between the temperature and the concentration of the nanoparticles with increasing thermophoretic effects. Although the error values vary, they are still small and near zero everywhere within the domain, indicating high numerical stability and convergence of the ANN framework. The error distributions obtained from the overall results confirm that the model LM-ANN is able to describe the nonlinear characteristics of both *Re* and *Nt* parameters with high accuracy and computational reliability for the passive transport mechanism.

## Conclusion

The heat and mass transfer properties of Casson nanofluid flow across a circular porous slider are examined in this work, taking into account the impacts of thermophoresis, Brownian motion, magnetic field and active-passive nanoparticle control. The governing equations are converted into a system of nonlinear ODEs using suitable similarity transformations. The numerical data are evaluated using RSM and LM-ANN approaches to examine parameter interaction, transfer phenomenon optimisation and to validate the thermal behaviour with the RKF-45. Thermal engineering systems, biomedical transfer procedures, cooling systems and porous surface applications may all benefit from the present research’s findings. An overview of the results from the current investigation is provided as follows:

- Higher values of *Re* increase the transverse velocity and change the profile of the vertical velocity and the effect is even greater with higher values of the non-Newtonian parameter *β *.
- With higher values of *β *, the non-Newtonian nature of the flow becomes dominant and makes the velocity profile more sensitive to changes in *Re*.
- Higher values of the magnetic parameter *M* decrease the primary and secondary velocity profiles due to the effect of resistance by the Lorentz force.
- The temperature profile becomes lower as the value of *Re* increases for both active and passive nanoparticle control cases. Inertial effects cause acceleration in the fluid flow.
- In the presence of active nanoparticles, an increase in the value of the Brownian motion parameter *Nb* results in a significant increase in the heat transfer. This is not the case when there are passive nanoparticles, since the zero net nanoparticle flux condition greatly limits the movement of nanoparticles in proximity to the wall.
- With increasing thermophoresis parameter *Nt*, the temperature profile increases in the case of active and passive control, respectively, due to increased nanoparticle movement and heat transfer.
- In comparing active and passive controls in terms of temperature profile, active control has greater sensitivity towards Brownian motion than passive control.
- ​ An increase in the value of *Nt* leads to an increase in the concentration profile, while an increase in *Nb*, *Sc* and *Kr* leads to a decrease in the concentration profile.
- An increase in the value of the Schmidt number *Sc* decreases the concentration due to decreased mass diffusivity. Similarly, an increase in the value of the *Kr* decreases the concentration due to increased reaction.
- The accuracy of the RSM model is around 99.48% for the active case and 99.88% for the passive case. Compared to the active example (0.120733), the passive case has a reduced standard error of regression (0.0337993).
- The ANN model is useful in nonlinear thermal-fluid transfer analysis because its prediction capacity is accurate, with minimal error values and findings consistent with the numerical simulation results.
- These results have applications in industry for porous slider bearings and rotating equipment with lubrication, where there is a need for controlling flow and heat transfer. Active or passive control of nanoparticles and magnetic field can also be applied in cooling systems, heat exchangers and microfluidic devices.

The current approach provides an excellent way to describe the simultaneous momentum, heat and nanoparticle transfer around the circular porous slider. One of the main strengths of this approach lies in its capability to explore the effect of magnetic field and nanoparticle activity on transfer phenomena. This approach is based on several assumptions related to the selected physical properties of the fluid flow, geometry and boundary conditions. The next step in the future could be the study of more complex porous structures with the variation of thermophysical properties and nanoparticle interaction.

## Data Availability

The findings of this work are based on theoretical analysis; no external data were required. Hence, all the data generated or analysed during this study are included in this article.

## Nomenclature

*Re*: Reynolds number
*M*: Magnetic field parameter.
*E*: Activation energy parameter
${K}_{r}$: Chemical reaction parameter
*\Pr *: Prandtl number
*(x,y,z)*: Direction
${k}_{r}^{2}$: Chemical reaction rate
$${C}_{p}$$: Specific heat
*k*: Thermal conductivity
*ρ *: Density
$$\theta (\eta )$$: Temperature profile
ANN: Artificial neural network
RKF: Runge-Kutta-Fehlberg
MHD: Magnetohydrodynamics
ANOVA: Analysis of variance
LM-ANN: Levenberg-Marquardt artificial neural network
AE: Absolute error
*Sc*: Schmidt number
$${N}_{t}$$: Thermophoresis parameter
${N}_{b}$: Brownian motion parameter
$${\theta }_{w}$$: Temperature ratio
*(u,v,w)*: Velocity components
*β *: Casson parameter
*T*: Temperature
*μ *: Dynamic viscosity
*σ *: Electrical conductivity
${B}_{0}$: Magnetic field strength
*ν *: Kinematic viscosity
RSM: Response surface methodology
ODEs: Ordinary differential equations
PDEs: Partial differential equations
CCD: Central composite design
MSE: Mean squared error
